# The complex genetics and biology of human temperament: a review of traditional concepts in relation to new molecular findings

**DOI:** 10.1038/s41398-019-0621-4

**Published:** 2019-11-11

**Authors:** C. Robert Cloninger, Kevin M. Cloninger, Igor Zwir, Liisa Keltikangas-Järvinen

**Affiliations:** 1 Department of Psychiatry, Washington University School of Medicine, St. Louis, MO USA; 2 School of Arts and Sciences, Department of Psychological and Brain Sciences, and School of Medicine, Department of Genetics, Washington University, St. Louis, MO USA; 3 Anthropedia Foundation, St. Louis, MO USA; 4 Department of Computer Science, University of Granada, Granada, Spain; 5 Department of Psychology and Logopedics, University of Helsinki, Helsinki, Finland

**Keywords:** Diagnostic markers, Personalized medicine

## Abstract

Recent genome-wide association studies (GWAS) have shown that temperament is strongly influenced by more than 700 genes that modulate associative conditioning by molecular processes for synaptic plasticity and long-term learning and memory. The results were replicated in three independent samples despite variable cultures and environments. The identified genes were enriched in pathways activated by behavioral conditioning in animals, including the two major molecular pathways for response to extracellular stimuli, the Ras-MEK-ERK and the PI3K-AKT-mTOR cascades. These pathways are activated by a wide variety of physiological and psychosocial stimuli that vary in positive and negative valence and in consequences for health and survival. Changes in these pathways are orchestrated to maintain cellular homeostasis despite changing conditions by modulating temperament and its circadian and seasonal rhythms. In this review we first consider traditional concepts of temperament in relation to the new genetic findings by examining the partial overlap of alternative measures of temperament. Then we propose a definition of temperament as the disposition of a person to learn how to behave, react emotionally, and form attachments automatically by associative conditioning. This definition provides necessary and sufficient criteria to distinguish temperament from other aspects of personality that become integrated with it across the life span. We describe the effects of specific stimuli on the molecular processes underlying temperament from functional, developmental, and evolutionary perspectives. Our new knowledge can improve communication among investigators, increase the power and efficacy of clinical trials, and improve the effectiveness of treatment of personality and its disorders.

## Introduction

Observers since antiquity have suggested that children are born with a natural disposition or style of how they react behaviorally and emotionally to diverse physiological, psychosocial, and energetic stimuli^1–3^. This innate biological disposition was called a person’s temperament and originally referred to a person’s animal-like nature as manifest in habitual patterns of automatic activity and emotional reactivity (temper)^1–6^. When measured in this traditional way, temperament is moderately stable on average throughout a person’s life span, but can be modified by behavioral conditioning^5–8^. Despite moderate stability, there is also substantial complexity in the development of temperament, including multi-finality (i.e., a particular profile of traits in early childhood may have different outcomes later) and equi-finality (i.e., different profiles of traits in early childhood may have the same outcome later)^9–12^.

In contrast, the other aspects of personality that were presumed since antiquity to distinguish humans from ancestral animals were collectively called a person’s character. Kant defined character as what people make of themselves intentionally^3^. Put another way, character is the self-regulatory aspect of personality—that is, the way a person shapes and adapts responses to ever-changing external and internal conditions^6^. These self-regulatory processes include the executive, legislative, and judicial functions necessary for mental self-government and self-actualization of identity^13^. When measured in this way, the self-regulatory aspects of personality develop in incremental steps across the life span as people learn episodically from their personal, social, and cultural experiences what goals and activities interest them and why some goals may be more valuable and fulfilling than others^13–15^.

Many modern scholars and researchers have suggested a variety of empirical ways to distinguish temperament from other aspects of personality^7,16–22^. Others prefer to lump all aspects of personality together in profiles or sets of linear factors, suggesting that adult personality is essentially a culturally conditioned expression of childhood temperament^23,24^ despite their modest and complex patterns of empirical association^10–14,25–29^. Nevertheless, temperament involves emotional drives that are irrational and vary quantitatively in strength, whereas the self-regulatory components of personality have several properties that qualitatively distinguish them from temperament, as summarized in Table 1. Temperament has traditionally been distinguished from other aspects of personality by observations about its neurobiology, appearance in infancy, distinctive styles of automatic behavioral and emotional reactions, absence of intentional self-control or self-awareness, stability across the life span, heritability, and/or the evolutionary conservation of underlying molecular processes^1–8,13–19,30–40^.

**Table 1:** Features traditionally used to distinguish temperament from character (i.e., other aspects of personality)

Component of Personality	Temperament	Character	References
Biology	Presumed to be strongly biologically determined by innate predisposition (“constitution”), and objectively related automatic behavioral and emotional reactions	Often suggested to be learned by experience, but such learning may be regulated by innate predispositions to learn in response to personal, social, and cultural experience and subjective processes in self-awareness	^1–7,18,19,30–34^
Behavior	Automatic activity & emotionality	Regulation of behavior by Goals and Values	^1–4^
Learning	Procedural (How)	Intentional (What) and Evaluative (When/Where/Why)	^1,2,5,6^
Emotion	Basic/primary (e.g., fear, happiness)	Differentiated/secondary (e.g., shame, compassion)	^18,30–34^
Development	Moderately stable from infancy onward	Appears after infancy and matures by succession of later steps into adulthood	^1–3,6,7,19^
Heritability	Strong & independent of social learning and culture	Either weak or strong, & influenced by social and cultural learning (norm-favoring)	^6,18,33,35,37^
Evolution	Temperament as habit learning is highly conserved in all animals	Intentional self-regulatory functions begin to be expressed as basic emotions and attachments in mammals and become well-developed in higher primates	^4,33,36,38,39,42^

Unfortunately, the various criteria suggested to define temperament do not overlap fully and can even contradict one another at times. For example, high heritability or developmental stability has each been used as a criterion for temperament, which leads to disagreements about how to define temperament because they do not identify the same individuals^7,8,41^. Development in infancy is another criterion used to identify components of temperament, but not all cognitive-behavioral features that develop in infancy involve patterns of automatic reactivity that are highly conserved in the evolution of all animals: in particular, some aspects of executive attention and effortful self-control emerged only late in evolution among great apes^42,43^ but begin to develop in early childhood and then mature in steps across the life span^17,19^. Nevertheless, some recent temperament theorists have included such self-regulatory functions as temperament on the basis of their being heritable and beginning to emerge in early childhood^17^, even though they do not satisfy the other traditional distinguishing features of temperament.

Such definitional inconsistencies have arisen in part for the convenience of investigators with expertise in working with particular methods and samples. For example, some temperament investigators focus on cross-sectional assessments of young children and focus on whatever is present in early childhood. Others do longitudinal studies and focus on developmental stability, while others study inheritance in family and twin studies.

In addition, temperament investigators have sometimes relied too heavily on simplistic dichotomies like nature versus nurture, biology versus learning, and genes versus environment. Such dichotomies are totally inadequate to describe the complex phenotypic, genotypic, and environmental architecture of human personality^25–27^. For example, human beings have three distinct systems of learning and memory that are strongly associated with different components of personality: associative conditioning (i.e., how we learn to react automatically, including classical and operant conditioning), intentionality (i.e., what we learn as goals to purposefully seek, including self-direction and social cooperation for personal or mutual benefit), and self-awareness (i.e., when, where, and why we learn, including autobiographical memory with imaginative shifts in perspective taking underlying science, art, and spirituality)^27,44–46^. Each of these systems of learning is dissociable from the others, and each is moderately heritable, related to distinct brain circuitry, and their integration across the life span involves strong gene-environmental interactions in adapting to a wide variety of physiological, psychosocial, and energetic stimuli^27^.

We hypothesized that the distinction between temperament and character was more likely to be specified by identifying which system of learning and memory underlies temperament, not whether temperament is due to nature (genes and biology) rather than nurture (environment and learning)^6,25,26^. Consequently, the effective translation of knowledge about temperament requires attention to the complex architecture of personality along with knowledge of its evolution and complex patterns of development in individuals^8,14,42,47–49^.

**Fig. 1:**
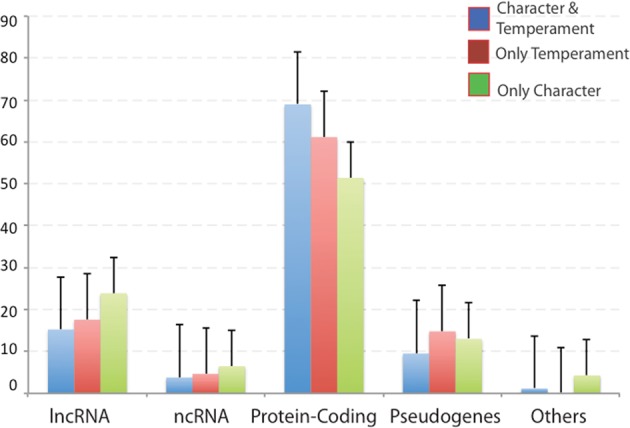
Distribution of biotypes of 972 genes associated with temperament and/or character, including long non-coding RNA (lncRNA), other non-coding RNA (ncRNA), protein-coding genes, pseudogenes, and others. Genes associated with temperament are more often protein-coding than those associated with character, which are more often genes with regulatory functions (lncRNAs and pseudogenes). Figure is reproduced from Fig. 4c of Zwir et al.^27^, Three Genetic-Environmental Networks for Human Personality)

Fortunately, we have recently used data-driven methods to identify single-nucleotide polymorphisms (SNPs) that map to 972 genes that explained nearly all the variability in temperament and character expected from twin studies in three independent samples of Finns, Germans, and Koreans^25–27^. These 972 genes include 245 associated with temperament only, 236 with character only, and 491 with both temperament and character. As shown in Fig. 1, the genes associated with temperament were more often protein-coding DNA genes than those associated with character^25–27^, which were more often long non-coding RNA genes or pseudogenes that influence the regulation of expression of protein-coding genes, coordination of the co-expression of sets of genes, and chromatin remodeling^50–52^. Most of the 736 genes associated with temperament are protein-coding genes involved in cellular processes of synaptic plasticity, associative conditioning, and related processes of stress reactivity and neurotransmission. The genes associated with personality were nearly always expressed in the brain (Supplementary Fig. 1). However, their brain functions frequently depended on interactions with genes for general housekeeping functions, such as the regulation of energy metabolism, cellular repair, and circadian rhythms, which occur in most or all cell-types and are associated with both temperament and character (Supplementary Fig. 1)^53,54^. These findings confirmed our hypothesis that the highly conserved molecular processes that regulate associative conditioning in experimental animals account for the heritability of human temperament. Our findings were confirmed in blindly independent replications by GWAS^25,26^ and by independent studies of gene expression during habit learning in experimental animals^25–27^.

However, all our studies were conducted using the Temperament and Character Inventory (TCI) in which heritable dimensions of temperament are assessed by scales that measure individual differences in disposition to associative conditioning in response to signals of punishment (i.e., Harm Avoidance: fearful, shy), novelty (i.e., Novelty Seeking: exploratory, impulsive-aggressive), signals of reward (i.e., Reward Dependence: attached, approval-seeking), and intermittent reinforcement (i.e., Persistence: determined, ambitious)^26^. Therefore, here we will review the relations of our temperament measures with alternative modern measures of temperament. We will also review the habitual patterns of behavioral activity and emotional reactivity to various physiological, psychosocial, and energetic stimuli expected from traditional concepts of temperament with those observed for the molecular pathways we uncovered for temperament (i.e., Ras-MEK-ERK and PI3K-AKT-mTOR pathways). Finally, we will discuss the research and clinical implications of our findings about the complex genetics and biology of temperament for translational psychiatry.

Our new molecular finding and this review of several complementary lines of temperament research provide an excellent opportunity to build consensus within the diverse field of temperament research and practice, which has been lacking^17,22^. We hope to clarify controversies among temperament researchers who use different assessment methods and study different groups of subjects without losing the complementary insights that may be derived from the different strategies that have been employed in studying temperament. Establishing a consensus in which complementary lines of research may help us all to translate the extensive work that has been and is being done into a more comprehensive model of human development could facilitate a more realistic understanding of many complex aspects of temperament and personality that are important for understanding and promoting healthy development.

## Partial overlap of concepts of temperament

Early descriptions of temperament focused on formal features of patterns of habitual behavioral activity and emotional reactivity that could be directly observed to response to environmental perturbations. In particular, seasonal variation in temperature (cold/hot) and rainfall (wet/dry) appeared to elicit individual differences in behavior and emotional reactions, and such observations gave rise to the ancient model of four temperament types^2,3,55^. These ancient temperament types were also distinguishable in terms of emotional style, valence of mood, intensity of arousal, and responses to rewards, novelty, and punishment, as summarized in Supplementary Table 1.

There has been no consensus about the optimal way to measure temperament based on these general distinguishing features, so a variety of measurement approaches have been used^7,12,17^. In order to relate the new molecular findings about the TCI to other models, we will first describe how the TCI measures temperament and then compare it to several alternative measures in order to assess the extent of their concordance conceptually and empirically.

### TCI measures of temperament

The Temperament and Character Inventory was developed as a neurobiologically-based model of the evolution of learning by extending the research of Jeffrey Gray on the relationship of associative conditioning in experimental animals to adult human personality^39,56^. Put another way, the TCI measures individual differences in behavioral and emotional style, which Thomas and Chess^5^ described as how a person acts automatically from disposition and habit, rather than the intentional and self-regulatory aspects of personality that specify what, when, where, or why they act as they do.

Specifically, the TCI measures four temperament dimensions that have been empirically confirmed to quantify individual differences in associative conditioning and related human brain circuitry: Harm Avoidance (i.e., fearful, pessimistic vs. risk-taking, optimistic)^57–59^, Novelty Seeking (i.e., impulsive, excitable vs. deliberate, reserved)^60,61^, Reward Dependence (i.e., sociable, sentimental vs. detached, objective)^58,61^, and Persistence (i.e., determined, ambitious vs. easily discouraged, underachieving)^62,63^. High and low scorers on all the subscales of TCI temperament and character are given in Supplementary Table 2 to help relate TCI variables to the terminology of other measures. Harm Avoidance is an indicator of negative valence that measures passive-avoidance learning and increased sensitivity to behavioral inhibition in response to fearful stimuli, which is mediated by activation of the amygdala, subgenual cingulate cortex, and the insular salience network^59,64,65^. Novelty Seeking is an indicator of positive valence that measures behavioral activity to approach and explore novel stimuli^66,67^, even if they do not predict rewards^61^. In contrast, Reward Dependence is characterized by social attachment and approach to rewards based on a different pattern of activation of dopaminergic neurons in the nucleus accumbens and substantia nigra from that seen in association with Novelty Seeking^61^ and on oxytocinergic neurons in the hypothalamus^68^. Persistence measures individual differences in rates of extinction of intermittently rewarded behaviors in response to frustrative non-reward, which is mediated by activating a brain circuit connecting the nucleus accumbens, anterior cingulate, and ventrolateral frontal cortex^62,63^. Furthermore, these brain circuits for behavioral conditioning are modulated by regulation of the co-expression of sets of genes in the two major molecular pathways for response to extracellular stimuli: the Ras-MEK-ERK pathway and the PI3K-AKT-mTOR pathways^25–27^, as will be described after reviewing the relations of the TCI to other ways of measuring temperament in children and adults.

### Strelau temperament inventory

Jan Strelau has produced a well-validated measure of temperament that is perhaps closest to the classical description of temperament by Kant^3,4^. Rather than speculating about temperaments being mixtures of various bodily fluids, Kant introduced the notion that temperaments could be recognized by observation of the formal characteristics of their behavior, which involve their energetic and temporal style rather than the content, situation, or goals of the behavior. Likewise Strelau observed that the most frequent and consistent indicators of temperament were its biological basis, presence since early childhood, appearance in both man and animals, and the formal characteristics of behavior as described by Kant^4,69^. Strelau found that the two formal characteristics of behavior emphasized by Kant (i.e., activity and emotional reactivity) had strong effects on the regulation of a person’s style and engagement in various behaviors and situations according to their stimulus value and psycho-physiological costs. Accordingly, his inventory, the Formal Characteristics of Behavior–Temperament Inventory (FCB-TI), measures self-reports of six formal characteristics of behavior in adults: Emotional reactivity (i.e., intense arousal), Briskness (i.e., quick tempo of response with mobility and flexibility), Sensory Sensitivity (i.e., low stimulus threshold), Activity (i.e., high energy level and social activity), Perseverance (i.e., persistence of action after cessation of reinforcing stimulation), and Endurance (i.e., tenacity despite long and intense stimulation). He has shown that his measures are moderately heritable and stable, and that they have strong correlations with other measures of temperament and personality, including the TCI, Pavlovian Temperament Survey (PTS), Buss and Plomin’s Emotionality–Activity–Sociability (EAS) inventory, the Eysenck Personality Questionnaire-Revised (EPQ-R)^4,69^, and the Revised Dimensions of Temperament Survey (DOTS-R) based on the features of behavioral style reported by Thomas and Chess to be moderately stable throughout childhood and adolescence^70^ (Table 2).

**Table 2:** Correlations (*r* × 100) of Strelau’s self-reports of basic energetic and temporal characteristics in adults with TCI (*n* = 282^69^ and other temperament surveys (*n* = 392^4^)

Strelau’s formal characteristics of behavior–Temperament Inventory						
Other inventories	Emotional Reactivity (intense arousal)	Briskness (quick tempo, mobility, flexibility)	Sensory Sensitivity (low stimulus threshold)	Activity (high energy and social activity	Perseverance (persistence after cessation of reinforcing stimuli)	Endurance (tenacity under long intense stimulation)
*TCI*						
Harm Avoidance	*73*	−*51*	**−16**	−*54*	*48*	−*57*
Novelty Seeking	**−22**	**18**	**16**	*40*	−1	6
Reward Dependence	**23**	−1	3	2	*36*	**−21**
Persistence	**−20**	**19**	10	**21**	1	**13**
Self-direction	−*40*	**29**	**16**	9	**−34**	**25**
Cooperation	−5	**18**	**19**	−11	−5	−4
Self-transcendence	7	0	6	**14**	12	−4
*Pavlovian TS*						
Strength of Inhibition	−**30**	**30**	7	5	−**23**	**39**
Strength of Excitation	−*57*	*49*	1	*37*	−*38*	*59*
Mobility	−*46*	*43*	11	**30**	−**28**	*46*
*EAS-TS*						
Distress	*59*	−*38*	−1	−**30**	*40*	−*43*
Fear	*53*	−*42*	−14	−25	*41*	−*43*
Anger	*40*	−**20**	7	1	**31**	−34
Activity	−20	**31**	7	*48*	2	**15**
Sociability	−**17**	**13**	−5	*47*	−8	6
*EPQ-R*						
Neuroticism	*72*	−*44*	2	−**21**	*59*	−*54*
Extraversion	−**32**	**27**	0	*73*	−11	**21**
Psychoticism	−**21**	5	−**13**	**16**	−**27**	8
*DOTS-R*						
Activity-general	−**8**	8	0	**29**	4	0
Activity-sleep	**13**	−6	2	0	12	−13
Approach vs. withdrawal	−**26**	**24**	**12**	*37*	−8	**20**
Flexibility vs. rigidity	−**33**	**32**	−6	**17**	**21**	**28**
Mood quality	−**17**	**18**	9	**26**	−7	**14**
Rhythmicity–sleep	5	3	−2	−6	1	−4
Rhythmicity–eating	−9	6	−7	6	−9	1
Rhythmicity–daily habits	7	9	−6	4	−2	−10
Low distractibility	**−20**	**22**	−**20**	10	−**13**	**21**
Persistence	**−14**	**15**	−**14**	−1	−6	**17**

Significant correlations are in bold (*p* < 0.05) or in italic (*p* < 0.01 plus *r* > 0.35)

TCI Harm Avoidance was the temperament most strongly correlated with the features of Strelau’s inventory, whereas none of the TCI character measures had strong correlations with the formal energetic and temporal characteristics of behavioral style. As shown in Table 2, Emotional Reactivity correlated strongly (*r* > 0.7) with TCI Harm Avoidance and EPQ Neuroticism, and moderately (0.7 > *r* > 0.35) with low scores on Pavlovian Mobility and Strength of Excitation and with high scores on negative emotions (distress, fear, anger) on the EAS. Activity is correlated strongly with EPQ Extraversion and moderately with TCI (low Harm Avoidance and high Novelty Seeking). Strelau’s formal characteristics were weakly correlated with DOTS-R measures of Thomas and Chess’s behavioral styles related to patterns of Adaptability (i.e., approach vs. withdrawal, flexibility vs. rigidity, mood quality) and Attentional Focus (i.e., low distractibility, persistence), but there were no significant correlations with Rhythmicity (i.e., regularity in sleep, eating, or other daily habits). Except for the weak correlations observed for Sensory Sensitivity, all of Strelau’s measures of the formal energetic and temporal characteristics of behavioral style have strong to moderate correlations with one or more TCI temperament traits and with factors in other tests, but not with TCI character traits. TCI temperament traits, but not character traits, also show rhythmicity, as discussed later.

The structural concordance of the four TCI temperaments with other measures of temperament has also been confirmed by their joint factor analysis with temperament as measured by the FCB-TI, EAS, and the DOTS-R in the Young Finns Study in 1997 when the 2106 participants were 20–35 years of age (Supplementary Table 3)^71^. Four factors corresponding to the four TCI temperaments were also identified by factor loadings over 0.5 of scales from the other tests: (1) Harm Avoidance along with FCB-TI Emotional Reactivity and EAS Negative Emotionality; (2) Reward Dependence along with EAS Sociability; (3) Novelty Seeking along with EAS and FCB-TI Activity; (4) Persistence along with DOTS-R Persistence. In addition, DOTS-R contained two factors with loadings over 0.5 that were not represented by the other tests: Rhythmicity (regularity in daily activities, sleep, and eating) and Flexibility, as was also observed by Strelau (Table 1).

### New York and Colorado surveys of childhood temperament

The Colorado Childhood Temperament Inventory is a parental report inventory designed to assess the temperament of children from ages 1 to 6 years. It was derived by factor analysis from the features identified by Thomas and Chess in the New York Longitudinal Study and those identified by Buss and Plomin in their original Emotionality–Activity–Sociability–Impulsivity (EASI) inventory^72^. Recent work shows that such parent reports are only slightly biased by the personality and mood of the parents^73^. Using parent interviews and direct observations of children, Thomas and Chess measured temperament in terms of nine dimensions that appear early in childhood: activity, rhythmicity, approach vs. withdrawal, adaptability, intensity of reaction, threshold of responsiveness, quality or valence of mood, distractibility, and attention span/persistence^5^, which were later adapted by Windle in the DOTS-R for adolescents^70^.

In contrast, Buss and Plomin measured temperament in terms of four behavioral factors that they found to be heritable and developmentally stable^7,18^. The two systems overlapped extensively, especially in indicators of sociability, emotionality, and impulsivity. Six temperament factors appeared in the original merger of the two systems^72^. From the EASI, Emotionality (i.e., easily distressed or irritated), Activity (i.e., highly energetic), and Sociability (i.e., easily approached, warmly responsive, prefers presence of friends to being alone) were retained, but EASI impulsivity was divided into two components called Persistence (i.e., persevering, long attention span) and Soothability (i.e., easily calmed and distracted from distress) based primarily on items from the New York Longitudinal Study (NYLS). A sixth factor, Reaction to Food, was also originally contributed by items from the New York Longitudinal Study^72^, but later a factor for Shyness (i.e., inhibited and fearful around strangers) from the EAS was substituted as a more general indicator of fearful, inhibited behavior^74^. Impulsivity was dropped when the EASI survey was modified to form the EAS survey (see Table 2) because their measure of impulsivity was made up of heterogeneous features that were not all heritable or present in infancy^18^. Nevertheless, both Persistence and Soothability were retained in the modified CCTI^48^. The four factors of Emotionality, Activity, Sociability, and Persistence were only weakly correlated with one another, whereas Soothability (*r* = −0.42) and Reaction to Food (*r* = 0.25) were correlated with Emotionality (*p* < 0.001)^72^.

Thomas and Chess observed that “temperament individuality” was established by 2 or 3 months of age in the NYLS^5^. They identified three temperament subtypes with specific profiles (i.e., configurations of characteristics) that were relatively stable from 2 months to 10 years of age, which they called “easy”, “difficult”, and “slow to warm up” subtypes^5,75–77^ (Supplementary Table 4). Others have confirmed that the structure and levels of temperament scales and profiles are moderately stable from ages 1 to 5, and stronger thereafter, using a variety of instruments including the CCTI, EASI, EAS, DOTS-R, and preschool TCI or ratings of temperament profiles based on direct observations in early childhood^28,48,78–80^. However, as previously mentioned, there is also substantial complexity in the development of temperament scales and profiles, including multi-finality and equi-finality^9–12,29^. The original three prototypes described by Thomas and Chess accounted for only 65% of children, but more advanced prototype matching and clustering methods allow classification of nearly all subjects^26,81^. As a result of the early classification problem, many investigators preferred to emphasize continuous measures of temperament, usually three linear factors corresponding to negative affectivity/neuroticism, positive affectivity/extraversion, and effortful control/conscientiousness, as in Rothbart’s Child Behavior Questionnaire and Early Adolescent Temperament Questionnaire^19^. However, the frequent emphasis on dimensions rather than prototypes may be questioned because of the greater stability of profiles compared to their component scales in complex adaptive systems and developmental studies of temperament^1,14^, the greater value of a profile of the whole person for therapeutic interventions^81^, and now our finding that genotypic influences on temperament are acting on multidimensional profiles, not the individual traits commonly measured in inventories^26^. Fortunately, measurement of multiple temperament dimensions allows both quantification of individual traits and classification of multidimensional prototypes.

The six CCTI temperaments have been found to have strong correlations with the TCI scales in preschoolers, as shown in Table 3^48^. The CCTI temperaments accounted for most of the variability in each of the four TCI temperaments (mR^2^ = 61–84%). However, CCTI scales did not significantly represent TCI Self-Transcendence (mR^2^ = 23%), which develops along with self-awareness later in childhood and adulthood. On the other hand, TCI scales did not significantly represent CCTI Activity (mR^2^ = 20%), which measures restless motor activity, even though they do for other scales of Activity that measure extraverted social activity or persistent and enduring activity (e.g., Table 1, Supplementary Table 3). The relationships between the TCI temperaments with parental reports of preschoolers were strong and similar to those observed with the formal characteristics of behaviors reported by adults in Strelau’s work: Harm Avoidance with CCTI Shyness (*r* = 0.82), Novelty Seeking with CCTI Emotional irritability (*r* = 0.64), Reward Dependence with CCTI Sociability (*r* = 0.74), and TCI Persistence with CCTI Persistence (*r* = 0.86). There were also weak to moderate correlations of TCI character traits with temperament as measured by TCI or CCTI, as expected due to the immature but developing functions of Self-directedness and Cooperativeness in self-regulation of Emotionality, Soothability, and Persistence.

**Table 3:** Correlations (*r* × 100) of preschool TCI with Colorado Childhood Temperament Inventory (CCTI) in parent reports on 64 children at age 30 months

CCTI Dimension	HA	NS	RD	PS	SD	CO	ST	mR^2^
Activity	−18	14	25	0	22	0	16	20
Emotionality	**57**	**64**	−36	−9	**57**	−**63**	−25	**63**
Shyness	**82**	**20**	−**57**	−8	−33	−26	−30	**77**
Soothability	−46	−**64**	**56**	4	**42**	**54**	13	**56**
Persistence	1	−37	12	**86**	**48**	**45**	29	**83**
Sociability	−**43**	−15	**74**	8	32	30	22	**61**
mR^2^	**76**	**61**	**72**	**84**	**56**	**57**	23	

Adapted from Constantino et al.^48^; statistically significant correlations are shown in bold

### Adult temperament and personality inventories

The TCI scales also have a distinct and consistent pattern of relations with inventories designed to measure temperament or personality traits reported to be heritable and neurobiologically-based in adults (Table 4). Data are available about the Adult Temperament Questionnaire (ATQ) of Evans and Rothbart^82^, the Zuckerman–Kuhlman Personality Questionnaire (ZKPQ)^83^, the Emotionality–Activity–Sociability (EAS) Temperament Survey^29^, the revised Eysenck Personality Questionnaire^83^, and the revised NEO Personality Inventory of Costa and McCrae^84^. The ZKPQ has measures of Emotionality (Neuroticism), Activity, Sociability, and Impulsive Sensation Seeking, so it is the alternative five-factor model for adults that is most similar in structure to the EAS and EASI temperament models for children and adults (see Table 4). These adult inventories also include self-regulatory components of personality with moderate to strong correlations with character traits of Self-directedness and Cooperativeness, such as ATQ effortful control, NEO agreeability/ATQ affiliativeness versus ZKPQ hostility, and NEO conscientiousness. Just as was observed with TCI temperaments in early childhood (Table 3), TCI temperaments in adulthood are closely related to other measures of adult personality traits, with correlations between 0.5 and 0.7: Harm Avoidance with measures of Neuroticism/Emotionality, Novelty Seeking with Impulsivity, Reward Dependence with Sociability/Affiliation/Extraversion, and Persistence with Activity/Conscientiousness. Although these relations are moderate to strong and indicate pervasive overlap in the overall content of different tests of temperament and personality, there are no simple one-to-one relations among the different tests, as can be seen in Tables 2–4 regardless of the number and content of factors or the age of subjects.

**Table 4:** Correlations (*r* × 100) in adults between TCI Scales and proposed measures of temperament or heritable personality dimensions derived by linear factor analysis

Scales of Temperament and Character Inventory							
Other inventories^a^	HA	NS	RD	PS	SD	CO	ST
*ATQ*							
Non-aggressive negative affect	**60**				−**51**	−24	
Aggressive negative affect	39				−**47**	−**49**	
Extraversion	−38	28	**57**	21	28	38	32
Orienting sensitivity		30				2	**31**
Affiliativeness			**47**			**52**	29
Effortful control	−37			**43**	**41**		
*ZKPQ*							
Neuroticism	**66**				−**49**		
Impulsive sensation seeking	−39	**68**	−20				28
Hostility			−27		−32	−**60**	
Sociability	−38	37	31				
Activity	−29			**46**	36		
*EAS-TS*							
Negative emotionality	**57**				−**53**	−30	
Activity	−31			29			
Sociability	−25		**45**			30	
*EPQ-R*							
Neuroticism	**59**				−**45**		
Extraversion	−**53**	**44**	23				
Psychoticism		**41**	−**45**	−29	−31	−**42**	
*NEO-PI-R*							
Neuroticism	**63**			−20	−**62**		
Extraversion	−**55**	**40**	**52**	**40**	25		22
Openness	−25	**43**	25				37
Conscience	−26	−34		**51**	**41**		
Agreeability		−23	**40**			**61**	20

Correlations over 0.4 in bold and other significant correlations over 0.2 shown

^a^Other inventories are Adult Temperament Scale (ATQ)^82^, Zuckerman–Kuhlman Personality Questionnaire (ZKPQ)^83^, Emotionality–Activity–Sociability Temperament Survey (EAS-TS)^29^, Eysenck Personality Questionnaire (EPQ-R)^83^, and Revised NEO Personality Inventory (NEO-PI-R)^84^

In summary, TCI temperaments are differentially associated with other specific temperament scales using a variety of inventories based on a variety of characteristics, including the formal characteristics of behavior, onset in early childhood, developmental stability, and/or a heritable and neurobiological basis. However, these traditional criteria have not enabled investigators to specify the structure and content of temperament in a way that is discrete and distinct from other aspects of personality. The architecture of temperament and personality does not have the linear structure that is unrealistically assumed by linear factor analysis and classical psychometric test theory: there are significant relations among multiple components of one test with multiple components of other tests, rather than simple one-to-one relations between components of tests that focus on different characteristics (e.g., on formal energetic and temporal characteristics, on developmental stability, or heritability) regardless of age (Tables 2–4).

As a result of the complex internal structure of multi-scale temperament and personality inventories, most widely used inventories with documented evidence of criterion-related validity perform poorly when their structure is evaluated by confirmatory factor analysis^85,86^. Even in adulthood, development is complex and non-linear with substantial evidence of both multi-finality and equi-finality, as is expected for the behavior of non-linear dynamical systems involving learning to adapt to ever-changing conditions^14,25–27,87^.

Both automatic and self-regulatory aspects of personality are heritable^41,88^ and some self-regulatory aspects of personality begin to develop in early childhood^19^. Consequently more fundamental features of temperament than heritability and early appearance are needed to distinguish temperament from other aspects of personality. Therefore we will now discuss new molecular findings about the qualitative differences in systems of learning that have emerged at different stages in the evolutionary line of ancestors of modern human beings^4,27,42,89^ and that may distinguish temperament from other personality traits in a fundamental way that satisfies all the traditional concepts^25–27,90^.

## Dispositions in habit learning as the fundamental basis of temperament

The temperament scales of the TCI were developed to measure specific constructs of associative conditioning based on data about the genetic structure of human personality in twins, phenotypic structure of habit learning by associative conditioning in humans and experimental animals, and the evolution of neurobiological mechanisms by which animals learn to adapt to changing conditions in their environment^91–94^. Initially the model was limited to temperament traits only, but has always included subscales to measure how broad dispositions are expressed in different situations^92^, as shown in Supplementary Table 2. Later observations revealed that people with any temperament profile could be healthy or unhealthy depending on character traits of Self-directedness, Cooperativeness, and Self-Transcendence, that were initially described on the basis of concepts from humanistic and transpersonal psychology^6,13,56^. We also found that temperament and character were equally heritable^41^, and hypothesized that they were distinguished by the distinct properties of brain networks that were equally heritable but involved in dissociable forms of learning and memory that had emerged at different times in the long evolutionary history of human beings: associative conditioning (i.e., classical and operant conditioning), Intentionality (i.e., self-directed and purposeful goal-seeking and cooperative behavior for mutual benefit), and Self-awareness (i.e., transpersonal or self-transcendent behaviors including creative imagination, mental time-travel, theoretical reasoning, and appraisal of values from a transpersonal perspective)^25–27,42,95–97^. Comparative analysis of neuroanatomy and emergent cognitive-behavioral functions in the ancestors of human beings suggested that temperament involved associative conditioning, which is highly conserved in all animals^42^. In contrast, brain functions for intentional self-regulation only emerged in higher primates, and self-awareness with creative capacities for art, science, and spirituality is present only in modern human beings^42,45,89,95,98^. These three brain networks normally interact in a coordinated manner^99–101^, but they are dissociable developmentally^45,46,102^ and functionally^45,100,101,103–106^.

A major limitation of earlier model-driven approaches to constructing temperament and personality inventories has been the tendency of people to fit their data to questionable assumptions of classical test theory using linear factor analysis. As we have just described in the prior section, the approach of fitting data to models has resulted in the failure to produce a consensus about how to measure temperament or how to distinguish it from other aspects of personality because people begin and end with different theories. Likewise, the tendency to fit genotypic data to models with the assumption that genes act independently of one another in the development of complex phenotypes like temperament and character is unrealistic because there is strong evidence of extensive gene–gene interaction for these traits^107^. In fact, prior estimates of gene–gene interaction in family studies of twins account for ~50% of the broad heritability of a variety of personality traits, with a range of 25–77%^108–112^, as summarized in Supplementary Table 5.

The model-driven approach to genome-wide association studies has failed to uncover the genotypic–phenotypic structure of complex traits and left most variability in complex traits unexplained by observed genotypes^107^. Instead of the estimates of 50% heritability of personality expected from twin studies, the heritability explained by SNPs has usually been ~10%, with a range of 0–21%^113–123^, as summarized in Supplementary Table 6. Therefore, we sought a data-driven method to test theories by fitting models to the data without restrictive and arbitrary theoretical assumptions.

We used a data-driven method called Phenotype–Genotype Many-to-many Relations Analysis (PGMRA) to identify SNPs that map to 972 genes that explained nearly all the variability in both temperament and character expected from twin studies in three independent samples of Finns, Germans, and Koreans^25–27^. Our machine learning approach^124,125^ uses the Non-Negative Matrix Factorization (NMF) method, which identifies multidimensional patterns within different types of data, such as quantitative or categorical phenotypes, genotypes, environmental variables, and/or voxels of neuroimages^25–27,126–128^. To uncover the natural genotypic–phenotypic architecture of a complex trait like temperament, PGMRA first dissects genome-wide data and uncovers a genotypic architecture composed of sets of SNPs shared by subsets of individuals (i.e., SNP sets), thereby allowing for complex genotypic information (such as gene–gene interaction and linkage disequilibrium) independent of any information about the phenotype. Next, phenotypic data are independently organized into natural sets of features, such as configurations of temperament traits shared by subsets of individuals (i.e., phenotypic sets); this allows for complex phenotypic interactions, such as heterogeneous temperament profiles, independent of any information about the genotype. Cross-matching of the two types of sets reveals multiple associations restricted to subgroups of individuals, thereby allowing for complex developmental phenomena, such as multi-finality and equi-finality. Other variable domains can also be integrated into the analysis, such as parental rearing, cultural influences, and other environmental exposures with or without measuring genotypes. The data-driven algorithm functions to extract and organize as much information as is available to increase the study power, so that moderately sized samples of people who are thoroughly assessed can be well powered^107,128^.

Our discovery sample was the Young Finns Study, an epidemiological study of 2149 healthy Finnish children followed regularly from 1980 (ages 3–18 years) to 2012 (ages 35–50)^129^. All subjects had thorough standardized genotypic, environmental, and phenotypic assessments, including administration of the Temperament and Character Inventory (TCI) in 1997, 2001, 2007, and 2012^8,129^. We replicated the results in two independent samples of 902 healthy adults from Germany^130^ and 1052 from Korea^131,132^ in which comparable genotypic and phenotypic features were available^25,26^. PGMRA was used to uncover the complex genotypic–phenotypic associations in the two replication samples (Germans and Koreans) independent of information about the discovery sample. The process used in the discovery sample was blindly and independently repeated in each replication sample without assuming homogeneity within or across samples^107^. We accounted for ethnicity in each sample by using the first three principal components for ancestral stratification of SNP genotypes^25,26^. Then matching of genotypic–phenotypic associations across samples was identified using parsimonious models that balance accuracy with model complexity, thereby avoiding over-fitting^133^. Models were learned independently in diverse samples to provide a stringent test of reproducibility despite complexity that might result from possible genetic, ethnic, cultural and environmental heterogeneity^107^.

We identified three clusters of people using the TCI temperament scales that measure individual differences in associative conditioning, behavioral activity, and emotional reactivity^26^. The three clusters corresponded closely to temperament clusters described by Thomas and Chess as “easy”, “difficult”, and “slow to warm-up”^76,77^. People in our “reliable” cluster resembled children with an “easy temperament” and adults who were conscientious extraverts because they were well-controlled in activity and were warm and calm emotionally. In other words, they were high in Reward Dependence (i.e., sentimental, friendly, approval-seeking), low in Novelty Seeking (i.e., deliberate, thrifty, orderly), low in Harm Avoidance (i.e., optimistic, confident, outgoing, and vigorous), and high in Persistence (i.e., determined). People in our “sensitive” temperament cluster resembled children with a “difficult temperament” and adults who are neurotic and unstable because they were under-controlled in activity and emotionally hypersensitive. Put another way, they were high in Harm Avoidance (i.e., pessimistic, fearful, shy, and fatigable), high in Novelty Seeking (i.e., impulsive, extravagant), and high in Reward Dependence (i.e., sentimental, friendly), so they frequently had approach-avoidance conflicts, rejection sensitivity, and disorganized attachments. People in our “antisocial” temperament cluster resembled children with a “slow to warm” temperament and adults who are socially detached, careless, and impulsive. That is, they were low in Reward Dependence (i.e., cold, detached, independent), low in Persistence (i.e., easily discouraged), and high in Novelty Seeking (i.e., extravagant, rule-breaking, but not inquisitive), which is frequently associated with maladaptive antisocial conduct.

We found 51 SNP sets that mapped to 736 gene loci and were significantly associated with one or more of the temperament sets. The neuronal functions and molecular processes associated with particular SNP sets and temperament profiles are shown in Table 5. Seventy-four percent of the identified genes were unique to a specific temperament profile, but 20 of the 51 SNP sets show substantial multi-finality (pleiotropy) in which at least 25% of carriers of the SNP set have different temperament profiles (Table 5). Such detailed data about the genotypic–phenotypic relations of temperament clusters are only available using the TCI. However, to facilitate consideration by readers familiar with other tests and to guide future investigation, the replicated TCI findings can be tentatively translated into profiles using scales measured by eight other major models of temperament and personality for which the relations with the TCI are known (see Supplementary Table S7). The genotypic sets distinguish people with distinct temperament profiles, so different descriptive models are hypothesized to capture the same clusters from various perspectives based on their correlations with the TCI: most traits in each system are at least moderately correlated with one or more TCI temperaments that differentiate the associated genotypic clusters.

**Table 5:** Neuronal functions and molecular processes associated with particular SNP sets and temperament profiles, including reliable (R), sensitive (S), and antisocial (A) profiles, and numbers of subjects and of genes mapped to each SNP set

Neuronal functions	SNP set	SNP set name	genes *n*	subjects *n*	Temperament profiles
Neuroplasticity	G_28_15	Estrogen neuroplasticity	29	101	S or A
	G_41_33	GPCR neuroplasticity	15	56	S
	G_28_10	WD/CDK neuroplasticity	8	46	R
	G_38_23	Sensory sensitivity	16	39	S
	G_30_28	Hippocampal synaptic plasticity	10	34	S
Long-term memory	G_12_1	Episodic memory	66	146	R
	G_7_3	Neurogenesis	128	133	S or A
	G_12_11	Ras-AKT interaction	4	105	R, S, or A
	G_31_8	Neurotrophin	60	54	S or A
Energy production	G_26_14	Glucose transport	25	46	S or A
	G_25_20	Fatty acid oxidation	3	33	R
	G_36_29	Electron transport	49	25	S
Cognitive flexibility	G_21_18	Cognitive flexibility	15	116	R or A
	G_38_17	MAPK memory enhancement	13	14	R
	G_5_3	Regulation pathways	2	172	R
Resistance to stress, injury, & aging	G_8_8	Global inositol/chemokine pathways	286	224	R
	G_12_8	Neuroprotection	111	173	R, A, or S
	G_16_15	Interleukin-2 neuroimmune response	7	94	A
	G_21_17	TGFβ resistance to aging	26	67	R
	G_33_33	TGFβ memory enhancement	13	49	R
	G_30_10	TNF-based resilience	6	47	R
	G_37_6	Methylation-based gene silencing	23	26	R
	G_20_2	Enhanced memory	18	25	R
Cholinergic neuromodulation	G_13_10	Cholinergic neuromodulation	17	148	R
	G_13_12	Acetylcholine biosynthesis	1	78	S or R
	G_21_16	Acetylcholine biosynthesis	1	37	S or A
	G_25_3	Acetylcholine biosynthesis	2	16	S or A
Fear conditioning	G_30_9	ERK-IP3-PKC stress interaction	52	69	S
	G_39_21	RGS negative emotionality	5	56	S
	G_41_37	PI3K-MAPK cognitive function	11	41	S
Stress reactivity	G_7_2	GPCR dysregulation	147	211	S or A
	G_9_2	Serotonin-cytokine interaction	11	140	S or A
	G_16_5	ERK-IP3-PKC stress memory	1	87	R
	G_14_12	Ras-based stress memory	22	83	A
	G_21_3	cellular senescence	39	60	S or A
	G_11_7	HPA stress reactivity	11	26	S or A
	G_33_4	ERK-PKA interaction	6	24	S or A
	G_38_38	Ion permeability	18	38	S
	G_22_6	Blood-brain barrier permeability	30	37	S or A
	G_42_39	Approach-avoidance conflict	11	19	S
Conditioning of dopaminergic activation	G_16_1	PI3K-based memory	11	108	A
	G_35_22	PI3K-based memory	5	43	S or A
	G_39_26	mTOR myelination	26	20	S or A
Conditioning of neuroexcitability	G_7_7	Olfaction	58	145	A
	G_13_3	ERK-conditioned impulsivity	21	95	S or A
	G_35_7	PI3K-based memory	12	32	A
	G_37_14	Neuroexcitability	12	21	A
	G_36_18	Brain RNA biosynthesis	4	19	A
Habit extinction	G_38_13	Glucuronidase habit extinction	7	60	R, A, or S
	G_19_3	Glucuronidase habit extinction	5	48	S
	G_40_5	Mannosidase habit extinction	3	16	A

Adapted from Zwir et al.^25^, Tables 1 and 3

73.6% of the 736 genes associated with temperament were unique to a single temperament profile: 266 with reliable, 236 with sensitive, and 40 with antisocial

Put another way, the different models that have been developed for measuring temperament are like dialects of a common language with inconsistent accretions from their variable integration with character traits during development. The inconsistencies between these dialects are minimized when they are restricted to the multidimensional configurations that are associated with the common genotypic language of temperament. For example, each model of temperament identifies highly Harm Avoidant people as neurotic introverts (i.e., people who are high in Neuroticism, Negative Emotionality, or Emotional Reactivity) and low in Extraversion. However, Extraversion (i.e., positive emotionality) is a complex composite of low Harm Avoidance, high Novelty Seeking, high Reward Dependence, and some contributions from character traits (Tables 2 and 3). Consequently, Extraversion can indicate any of these TCI traits depending on its configurations with low Neuroticism (indicating low Harm Avoidance), high Impulsivity (indicating high Novelty Seeking), and/or high Sociability (indicating high Reward Dependence). Different inventories measure what they call high Activity in qualitatively different ways: it may involves extraverted social activity (i.e., high Reward Dependence, as in the Strelau FCB inventory), persistent and enduring activity (i.e., high Persistence, as in the ZKPQ or the Strelau FCB Inventory), or restless motor behavior, as in the EAS or CCTI, which is not consistently associated with TCI temperaments (see Tables 2 and 3). Like restless motor activity, TCI character traits are variably associated with TCI temperaments, as are ZKPQ Hostility and NEO Agreeability. Rothbart’s CBQ effortful control and NEO conscientiousness are composites of high TCI Persistence plus the self-regulatory character trait measured by high Self-directedness in the TCI. There is certainly loss of specificity for genotypic associations with such heterogeneous measures, but the multidimensional profiles should provide a useful tool for investigators without access to genotypic data about their own model, or for investigators to test the robustness of our genotypic findings with other models. We hope this information will also encourage more work with both individual dimensions and multidimensional profiles, which have complementary utility.

**Fig. 2:**
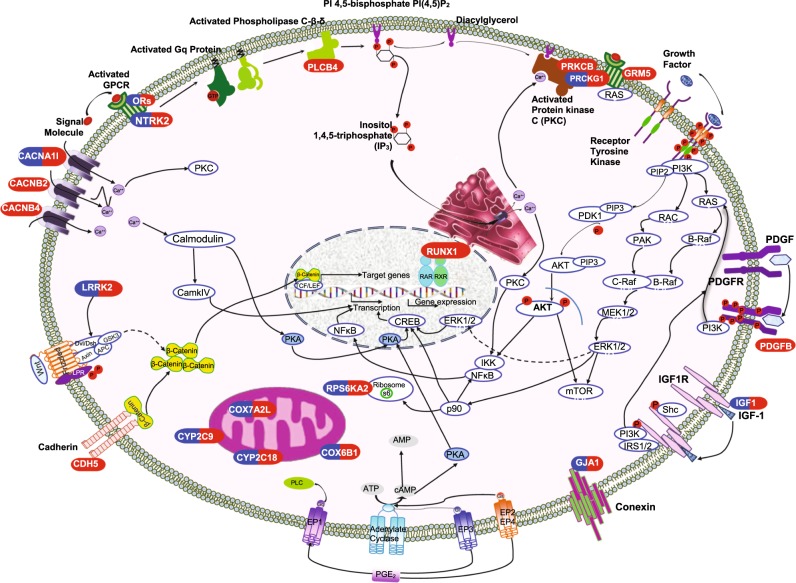
Cell displaying the molecular pathways containing genes associated with human temperament as measured by the Temperament and Character Inventory. The genes influence the Ras-MEK-ERK (MAPK), PI3K-AKT-mTOR, and Protein Kinase A, B, C pathways that regulate associative conditioning (reproduced from Fig. 2c of Zwir et al.^25^, Uncovering the Complex Genetics of Human Temperament)

What then is the common genotypic language of temperament? Most of the identified genes were enriched in pathways activated by associative conditioning in animals, including the ERK, PI3K, and related protein kinase pathways, which are highly conserved in all animals (Fig. 2). When activated, the Ras-MEK-ERK cascade (also known as the mitogen-activated protein kinase (MAPK) pathway) and the PI3K-AKT-mTOR cascade serve as the major cellular mechanisms for response to extracellular stimuli, including activation of processes that promote synaptic plasticity, associative conditioning, and long-term memory^134–138^. The cell-surface receptors for these pathways can be activated by a wide variety of physiological, psychosocial, and energetic stimuli that vary in positive and negative valence and in consequences for health and survival^136,139,140^. Changes in these pathways in response to associative conditioning occur in a coordinated manner with related processes including stress reactivity^141^, neuronal and glial growth^142^, and neurotransmission^143^. Both pathways converge on mTOR, which allows modulation of their joint action and circadian rhythmicity (Fig. 2)^26,144,145^.

In summary, our findings suggest that individual differences in associative conditioning (habit learning, including classical and operant conditioning) may be the fundamental molecular mechanism for human temperament. Individual differences in associative conditioning provides a precise definition and causal mechanism that accounts for all the traditional concepts about temperament being distinguished from other aspects of personality by its formal behavioral style (how we learn) and emotional reactivity, which correspond to response patterns that are highly conserved in all animals, present in people from early childhood, and moderately stable across the life span.

However, these findings also open up many more questions for future study by investigators with diverse interests and skills. Temperament researchers have varied in whether they focused on individual traits or subtypes defined by profiles of multiple traits. We found that most of the genes for each associated with each temperament subtype were unique to that subtype, which suggests that the natural unit of measurement of temperament are profiles of multiple traits within an individual, not single traits that differ between individuals^90^. Nevertheless, activation of the genotypic sets leads to different behavioral responses in response to different environmental challenges, so this needs to be considered, as we begin to describe in the next section. The same person can carry multiple genotypic sets, so their individual traits may be a mixture of the effects of these multiple genotypic sets, as we have described elsewhere^25,26^ along with vignettes of the pure prototypes^27^. In addition, environmental influences during development can influence the development of temperament substantially by influences on the way the antecedents of temperament and character become integrated and self-actualized, as we have also begun to explore^27^. Identifying the fundamental molecular mechanisms underlying temperament is expected to help move its investigation forward in a more integrated way, and opens up many opportunities for translational research and practice. We will illustrate some basic questions that need more thorough study by available results regarding stimuli that allowed observers to recognize the distinguishing features of temperament in antiquity and that may still guide us in developing interventions to facilitate the healthy functioning of people by understanding their temperament.

## Psychobiological modulation of temperament-related molecular pathways

Observations of temperament provide a direct window by which we can observe the powerful mechanisms that evolved in animals to allow rapid and effective adaptations to extracellular stimuli that are essential for the health and survival of all animals. Temperaments evolved in ways that help animals to adapt to naturally occurring variation in external and internal stimuli, which is essential for cellular proliferation and plasticity, resistance to degenerative processes (related to stress, injury, and aging), regulation of immune and inflammatory response, and maintenance of energy production, in addition to processes that mediate habit learning, emotional reactivity, cognitive flexibility, sensory sensitivity, and circadian rhythmicity, as shown for the functions of temperament-related SNP sets in Table 5^26,146–148^. Consequently the molecular mechanisms underlying temperament may play important roles in susceptibility to the common diseases that burden modern society as a result of direct expression in particular organs and as a result of indirect influences mediated by lifestyle behaviors^26,147–153^.

The effects of key physiological and energetic extracellular stimuli on the Ras-MEK-ERK and PI3K-AKT-mTOR cascades related to temperament are summarized in Table 6. These stimuli are important regulators of adaptive responses to diurnal, seasonal, and climactic variation in conditions that require automatic adaptation in order to maintain cellular homeostasis, healthy functioning, and repair mechanisms. These stimuli correspond to changing diurnal and seasonal conditions to which animals must adapt for their health, reproduction, and survival despite changes between hot and cold temperatures^154–158^, light and dark luminosity^145,159,160^, and other conditions including exposure to electromagnetic fields^161–168^, variable supplies of water^169–172^ and nutrients^172^, and variable demands for physical activity^173–175^ and opportunities for sleep^176–178^. Under experimental or natural conditions, diurnal and seasonal rhythmicity in activity is associated with individual differences in TCI temperaments: people who are high in Novelty Seeking prefer to be more active late at night rather than in the morning^179–181^ and are more likely to have been born during the long photoperiod of summer than the short photoperiod of winter^181,182^. Furthermore, diurnal rhythms in activity are associated with seasonal rhythms in activity, emotionality, sociability, and body temperature^180^, much like the descriptions of distinguishing features of the classical temperament subtypes (Supplementary Table 1).

**Table 6:** Effects of physiological and energetic extracellular stimuli on temperament-related Ras-ERK (MAPK) and PI3K-AKT-mTOR pathways

Extracellular stimulus	Effect on Ras-ERK	Effect on PI3K-AKT-mTOR	Cellular response	References
*Temperature*				
Cold	Inhibition		Quiescence (cold slows growth and metabolism, promotes repair of injury, reduces pain and inflammation)	Hypothermic stress^154^ repair of injury^155^
Hot		Activation	Growth and proliferation (heat increases growth and switches cells from catabolic to anabolic processes)	Heat stress^156,157^ anabolic switch^158^
*Luminosity (visible light)*				
Dark		Inhibition	Slows and dampens circadian rhythmicity via mTOR	Night ^145^
Bright	Activation	Activation	Accelerates and enhances circadian rhythmicity via mTOR, directs neurite outgrowth via Ras-ERK	Visible light pulses^145,159,160^
*Electromagnetic fields*				
External high frequency (not protected)	Inhibition	Inhibition	Exposure to non-thermal high-frequency EMF impairs hippocampus function, emotional stability, passive-avoidance learning, and regulation of impulse-control via inhibited Ras-Erk, and inhibited AKT and voltage-gated calcium channel signaling of self-control	External non-thermal GHz EMF exposure^161,162^
External high frequency (protected)			Administration of melatonin and omega-3 fatty acids protects against the harmful effects of non-thermal high-frequency EMF	Neuroprotection from non-thermal EMF^163^
Low-intensity and low-frequency EMF	Inhibition or activation		Exposure to low-intensity, frequency-modulated EMF can inhibit or activate depending on frequency, site, and temperament. 24 HZ EMF inhibits cell proliferation by inhibiting Ras-ERK (MAPK) pathways. In contrast, 10 HZ transcranial magnetic stimulation of dorsolateral PFC reduces negative affect in ways related to temperament and ERK pathway (uncoupling subgenual ACC from default mode network is reduced by higher Harm Avoidance, and increased by higher Persistence). Anti-depressant effects involve activation of Ras-Erk with proliferation of hippocampal-derived neural stem cells)	Frequency-modulated 10–25 HZ EMF exposure^164–168^
*Hydration*				
Dry	Inhibition		Dehydration inhibits components like AMPK and TSC around mTOR signaling, thereby reducing cellular energy from glucose intake, glycogen synthesis, lipogenesis, and ERK expression	Hyper-osmotic dehydration^169,170^
Wet	Activation		Hydration promotes Ras-ERK and mTOR signaling, increasing cellular energy availability	Hypo-osmotic hydration^171,172^
*Nutrition*				
Fasting		Inhibition by nutrient and energy depletion	The mTOR complex depends on nutrient availability so its activity is reduced by diverse mechanisms of energy depletion	Nutrient sensing by mTOR^172^
Feeding		Activation by various nutrients, particularly amino-acids, insulin-and growth-factor signaling		Nutrient sensing by mTOR^172^
*Exercise*				
Inactive	Low activity	Low activity		
Active	Activation	Activation	Exercise activates both ERK and mTOR signaling via increased expression of AMPK, CAMK4, and p38 genes, leading to increased cellular growth, energy availability from mitochondrial biogenesis in multiple body tissues, including neurons and muscle, and increased morphological plasticity of muscle and increased insulin sensitivity in diabetes and obesity.	Endurance training^173–175^
*Sleep*				
Deprived	Inhibition		Sleep deprivation reduces expression of Ras-ERK pathway, leading to impaired learning and memory, as observed in parasomnias associated with increased Novelty Seeking	Sleep deprivation^176,177^
Unlimited	Activation		Duration of sleep is regulated by ERK pathway by effects on expression of activity-dependent neuromodulators like norepinephrine during wakefulness	Modulation of sleep and wakefulness^178^

## Translating the new genetics of temperament for research and practice

The first and major implication of the new genetic findings is a precise definition of temperament, which is really a fundamental need for good communication and incremental research progress within any scientific field. Based on the findings reviewed here, we propose the following definition: *Temperament is the disposition of a person to learn how to behave, react emotionally, and form attachments automatically by associative conditioning (that is, rapidly and spontaneously, without conscious attention or reflection in response to changing internal and external conditions)*. Each part of the definition outside the explanation in parenthesis is essential: (1) temperament is the organization within an individual (i.e., a disposition, or set of distinguishing features) of how a person learns, not what, when, where, or why they learn; it involves the form and style of how a person learns; (2) the characteristic features involve what can be learned by associative conditioning, which include habitual patterns of behavior, emotional reactions, and attachments; (3) learning by associative conditioning in response to changing conditions is automatic and spontaneous (that is, without delay for conscious attention or reflection).

We propose that these criteria are necessary and sufficient to define temperament precisely. Our proposed definition is sufficient because it implies all the traditional criteria proposed for temperament, and it is necessary because the other criteria are non-specific when used individually or in combination. From this basic definition, it follows that the predisposition to temperament is innate and heritable, but its expression may change in response to associative conditioning, which can be modified by brain development or injury and by its integration with other systems of learning and memory related to other aspects of personality involving self-regulatory processes for intentional self-control and creative self-awareness. Associative conditioning is highly conserved in all animals, whereas intentional self-control emerged only in higher primates and self-awareness in human beings^25–27^. The integration of these systems is manifest in the complex and dynamic patterns of development that are observed for personality, language, art, and science across the life span of a person in response to changing conditions^27^.

We suggest that the proposed definition of temperament captures all the traditional concepts with specificity and precision, distinguishing it from other aspects of personality with which it becomes integrated during development. For example, a temperament can be unambiguously distinguished by heritable differences in behavioral conditioning; what is inherited as temperament is limited to the habit learning system, the component of procedural learning that is evolutionarily conserved in all animals. Cognitive systems for intentional self-control that emerged in higher primates may begin to interact with temperament from an early age^19^, but they involve fundamentally distinct molecular processes and brain structures than does temperament^42,43^. This definition yields the expected features of appearance in early childhood, prominence of basic emotions and automatic behaviors, and moderate stability over time, which also distinguish temperament from other aspects of personality, as summarized in Table 1.

An alternative definition is that “temperament refers to neurochemically based individual differences in the regulation of formal dynamical aspects of behavior^22^.” Reference to the formal dynamical aspects of behavior, as did Strelau (see Table 2), is useful to exclude character, but does not capture the rhythmicity and responsiveness to physiological stimuli (e.g., hot/cold, wet/dry, light/dark) that is prominent in classical concepts of temperament (Supplementary Table 1), the prominence of social attachments (sociable/aloof) (Tables 2–4), or in the molecular processes for regulation of diurnal and seasonal rhythms that we identified as fundamental features of the molecular pathways underlying temperament (Tables 2 and 6). We propose that only the form of learning (i.e., associative conditioning) and its evolutionary conservation are necessary and sufficient criteria for temperament because of the non-specificity of other criteria.

Several traditions that have approached temperament in different ways^17^ can now be recognized as converging and providing complementary information about how temperament and other aspects of personality develop across the life span. Defining temperament in terms of a specific and heritable form of learning makes it clear that distinctions between nature and nurture, biology and learning, genes and environment are inadequate. Temperament is the manifestation of a specific form of learning and memory, which is a non-linear dynamical process associated with complex patterns of inheritance and development. Individual differences in these adaptive processes are being investigated in terms of specific human brain functions using brain-imaging techniques^96,97,126,183^.

The temperament and character domains of personality do not function independently, so it is not surprising that investigators interested in temperament or personality often address similar questions. At times the overlap and interaction of temperament and character has led to confusion about what belongs to which domain because people function as whole organisms embedded in the world. We have identified the networks that integrate these domains and described their architecture, but there remains a need for further research to understand the integrative processes that bring the emotional reactivity of temperament together in balanced way with emotional regulation of character.

Personality research has closely aligned itself with temperament research by its emphasis on stability and use of similar methods based on assumptions of linear structure. However, it is crucial to recognize that personality has a complex biopsychosocial structure that is a product of interactions among multiple systems of learning memory that are dissociable functionally and developmentally.

Our findings about the complex genetics of temperament and character can best be understood from an evolutionary-developmental perspective. The evolutionary-developmental perspective helps to understand the adaptive functions of the molecular processes that distinguish temperament from other aspects of personality. The functions of the Ras-MEK-ERK and PI3K-AKT-mTOR pathways serve to maintain cellular homeostasis, healthy functioning, and repair of injury and degeneration despite diurnal, seasonal, and climactic changes in a person’s internal and external environment. Diverse stimuli can activate the molecular systems underlying temperament in coordinated ways that provide opportunities for effective interventions. However, there is great need for clinical trials to clarify how to use these natural stimuli effectively. As we begin to recognize that the psychobiological and genetic networks that regulate health and well-being correspond to systems of learning and memory, we have the opportunity and responsibility to develop and advocate an evidence-based approach to psychiatry that integrates knowledge about molecular, neurobiological, and psychosocial processes. The molecular aspects of psychiatry are only one level of organization that helps to open our eyes to the full multi-level organization of human functioning.

We have found that combining genotypic and phenotypic information does provide more information about health than does phenotypic information alone^25,26^. Consequently, genotypic panels for assessing the health propensities of people based on their personality are likely to be developed and offered commercially, as is being done for complex medical disorders. However, what has not been acknowledged by such commercial ventures is that the development of common disorders is highly complex and depends on the interaction of many sets of genotypic and environmental variables. Polygenic risk scores are not adequate for precise assessment of temperament because they rely on the average effects of genes acting independently, which can provide only weak and inconsistent information about personal health or risks of complex phenotypes in a specific individual (Supplementary Table 6)^107^. Even when complex phenomena (i.e., pleiotropy, epistasis, and gene-environment interaction) are taken into account, it turns out that the same genotypic profiles can be expressed in ways that are either healthy or unhealthy because of differences in the coherence of processes that regulate expression of genes and co-expression of sets of genes, often involving long non-coding RNA genes or a few “switch genes” that distinguish healthy and unhealthy character profiles^25,27^. For example, every possible TCI temperament profile can be either healthy or unhealthy, depending on a person’s character profile; there are average differences in risk between profiles, but nothing can be said about how healthy a particular individual is from their temperament alone^14^. Until we learn more about the processes that regulate the expression of protein-coding genes^27^, the additional costs and worries introduced by genetic testing of personality and/or common diseases may be unjustified when most information of practical value for personalized treatment planning is provided by improved phenotypic assessment at a lower cost. In addition, there are serious ethical issues concerning germline editing of the human genome to modify heritable human traits^184^. Our current reservations about the merits and dangers of introducing genotypic panels for enhanced personality assessment will need to be revisited once we gain more knowledge about the regulation of co-expression of sets of genes that lead to well-being and ill-being.

Psychopharmacology has already made substantial advances in developing treatments designed to target specific receptors, which can be an effective strategy when a small number of receptors cause a disorder consistently. However, when heterogeneous disorders depend on complex interactions among many genes and environmental variables, it is difficult or impossible to design interventions that are broadly effective and well tolerated.

Fortunately, we already know that the molecular mechanisms underlying temperaments evolved to help organisms adapt to naturally occurring physiological, psychosocial, and energetic stimuli, as was observed in antiquity. What is most important now is to consider how our molecular and clinical observations can be translated into useful interventions for disease reduction and health promotion. Use of cold (e.g., cryotherapy)^185,186^, heat (e.g., infrared light therapy)^187^, light exposure (e.g., bright light therapy)^188,189^, patterned EMF (e.g., transcranial magnetic stimulation)^167^, and lifestyle adjustments to optimize hydration, nutrition, exercise, and sleep^190,191^ have been widely advocated, but often produce weak and inconsistent results, particularly when there is inadequate motivation for change^192^ or limited understanding of the underlying mechanisms and the parameters critical for efficacy^193,194^.

Furthermore, there is extensive evidence that treatments of temperament are most effective when treatment addresses all three systems of learning and memory in a coordinated manner: behavioral conditioning, intentional self-control, and self-aware evaluation need to be integrated in order to be strongly and consistently effective in promoting health and well-being^27,190,195–198^. Put another way, relating a person’s current well-being to both their temperament and character provides powerful motivation for a person to change^199^. Fortunately, such thorough phenotypic assessments can also be expected to improve clinical trials by increasing study power in moderate-sized samples with stronger and more consistent results than have been obtained in poorly characterized and heterogeneous groups of subjects^107^.

## Supplementary information

Supplementary Information

## References

[1] Kagan, J. *Galen’s Prophecy: Temperament in Human Nature*. (Basic Books, 1998).

[2] Avicenna. *The Canon of Medicine*. 1–710 (KAZI Publications, 1999).

[3] Kant, I. *Anthropology from a Pragmatic Point of View*. 1978 edn (Southern Illinois University Press, 1797).

[4] Strelau J (1996). The regulative theory of temperament: current status. Personal. Individ. Differences.

[5] Thomas, A. & Chess, S. *Temperament and Development*. (Brunner/Mazel, 1977).

[6] Cloninger CR, Svrakic DM, Przybeck TR (1993). A psychobiological model of temperament and character. Arch. Gen. Psychiatry.

[7] Goldsmith HH (1987). Roundtable: what is temperament? Four approaches. Child Dev..

[8] Josefsson K (2013). Maturity and change in personality: developmental trends of temperament and character in adulthood. Dev. Psychopathol..

[9] Cicchetti D, Rogosch FA (1996). Equifinality and multifinality in developmental psychopatology. Dev. Psychopathol..

[10] Moffitt TE, Caspi A, Harrington H, Milne BJ (2002). Males on the life-course-persistent and adolescence-limited antisocial pathways: follow-up at age 26 years. Dev. Psychopathol..

[11] Rettew DC, Althoff RR, Dumenci L, Ayer L, Hudziak JJ (2008). Latent profiles of temperament and their relations to psychopathology and wellness. J. Am. Acad. Child Adolesc. Psychiatry.

[12] Rettew DC, McKee L (2005). Temperament and its role in developmental psychopathology. Harv. Rev. Psychiatry.

[13] Cloninger, C. R. *Feeling Good: The Science of Well-being*. (Oxford University Press, 2004).

[14] Cloninger CR, Svrakic NM, Svrakic DM (1997). Role of personality self-organization in development of mental order and disorder. Dev. Psychopathol..

[15] Vaillant GE, Vaillant CO (1990). Natural history of male psychological health, XII: a 45-year study of predictors of successful aging at age 65. Am. J. Psychiatry.

[16] Dyson MW (2015). The structural and rank-order stability of temperament in young children based on a laboratory-observational measure. Psychol. Assess..

[17] Shiner RL (2012). What is Temperament Now? Assessing progress in temperament research on the twenty-fifth anniversary of Goldsmith et al. (1987). Child Dev. Perspect..

[18] Buss, A. H. in *Explorations in Temperament: International Perspectives on Theory and* Assessment (eds Strelau, J. & Angleitner, A.) Ch. 3, 43–60 (Plenum Press, 1991).

[19] Rothbart MK (2007). Temperament, development and personality. Curr. Directions Psychological Sci..

[20] Trofimova I, Robbins TW (2016). Temperament and arousal systems: a new synthesis of differential psychology and functional neurochemistry. Neurosci. Biobehav Rev..

[21] Trofimova I (2010). An investigation into differences between the structure of temperament and the structure of personality. Am. J. Psychol..

[22] Cloninger C. Robert, Zwir Igor (2018). What is the natural measurement unit of temperament: single traits or profiles?. Philosophical Transactions of the Royal Society B: Biological Sciences.

[23] McCrae RR (2000). Nature over nurture: temperament, personality, and life span development. J. Pers. Soc. Psychol..

[24] Kohnstamm, G. A., Halverson, C. F. J., Mervielde, I. & Havill, V. L. in *Personality and Clinical Psychology Series* (ed. Weiner, I. B.) 222 (Lawrence Erlbaum Associates, Mahwah, NJ, 1998).

[25] Zwir, I. et al. Uncovering the complex genetics of human character. *Mol. Psychiatry*, 10.1038/s41380-018-0263-6 (2018). 10.1038/s41380-018-0263-6 PMC7515844 30283034

[26] Zwir, I. et al. Uncovering the complex genetics of human temperament. *Mol. Psychiatry*, 10.1038/s41380-018-0264-5 (2018). 10.1038/s41380-018-0264-5 PMC7515831 30279457

[27] Zwir, I. et al. Three genetic-environmental networks for human personality. *Mol. Psychiatry*, 10.1038/s41380-019-0579-x (2019). 10.1038/s41380-019-0579-x PMC8550959 31748689

[28] Caspi A (2003). Children’s behavioral styles at age 3 are linked to their adult personality traits at age 26. J. Pers..

[29] Pesonen AK, Raikkonen K, Keskivaara P, Keltikangas-Jarvinen L (2003). Difficult temperament in childhood and adulthood: continuity from maternal perceptions to self-ratings over 17 years. Personal. Individ. Differences.

[30] Goldsmith, H. H. & Campos, J. J. in *The Development of Attachment and Affiliative Systems* (eds Emde, R. N. & Harmon, R. J.) 161–193 (Plenum, 1982).

[31] Ekman P (1992). Are there basic emotions?. Psychol. Rev..

[32] Sauter DA, Eisner F, Ekman P, Scott SK (2010). Cross-cultural recognition of basic emotions through nonverbal emotional vocalizations. Proc. Natl Acad. Sci. USA.

[33] Plutchik R (2001). The nature of emotions. Ameican Scientist.

[34] Izard CE (2007). Basic emotions, natural kinds, emotion schemas, and a new paradigm. Perspect. Psychol. Sci..

[35] Loehlin, J. C. *Genes and Environment in Personality Development*. (Sage Publications, 1992).

[36] Bouchard TJ, Loehlin JC (2001). Genes, evolution, and personality. Behav. Genet.

[37] Loehlin JC, Gough HG (1990). Genetic and environmental variation on the California Psychological Inventory vector scales. J. Pers. Assess..

[38] Darwin, C. *The Expression of The Emotions in Man and Animals; with an introduction, afterword and commentaries by Paul Ekman; Essay on the history of the illustrations by Phillip Prodger*. Ekman’s edition edn (HarperCollins Publishers, 1998).

[39] Cloninger CR (1994). The genetic structure of personality and learning: a phylogenetic model. Clin. Genet.

[40] MacLean PD (1985). Brain evolution relating to family, play, and the separation call. Arch. Gen. Psychiatry.

[41] Gillespie NA, Cloninger CR, Heath AC, Martin NG (2003). The genetic and environmental relationship between Cloninger’s dimensions of temperament and character. Personal. Individ. Differences.

[42] Cloninger CR (2009). The evolution of human brain functions: the functional structure of human consciousness. Aust. N.Z. J. Psychiatry.

[43] Allman JM (2011). The von Economo neurons in the frontoinsular and anterior cingulate cortex. Ann. N. Y Acad. Sci..

[44] Tulving E (2002). Episodic memory: from mind to brain. Annu Rev. Psychol..

[45] Tulving E (1987). Multiple memory systems and consciousness. Hum. Neurobiol..

[46] Levine B (2004). Autobiographical memory and the self in time: brain lesion effects, functional neuroanatomy, and lifespan development. Brain Cognition.

[47] Josefsson K (2013). Parental care-giving and home environment predicting offspring temperament and character traits after 18 years. Psychiatry Res..

[48] Constantino JN, Cloninger CR, Clarke AR, Hashemi B, Przybeck T (2002). Application of the seven-factor model of personality to early childhood. Psychiatry Res..

[49] Zohar AH, Zwir I, Wang J, Cloninger CR, Anokhin AP (2019). The development of temperament and character during adolescence: the processes and phases of change. Dev. Psychopathol..

[50] Fang Y, Fullwood MJ (2016). Roles, functions, and mechanisms of long non-coding RNAs in cancer. Genomics Proteom. Bioinforma..

[51] Quinn JJ, Chang HY (2016). Unique features of long non-coding RNA biogenesis and function. Nat. Rev. Genet.

[52] Barbash S (2017). Neuronal-expressed microRNA-targeted pseudogenes compete with coding genes in the human brain. Transl. Psychiatry.

[53] Faraone SV (2017). The omnigenic model: implications for psychiatric genetics. J. Psychiatry Brain Sci..

[54] Hess JL, Akutagava-Martins GC, Patak JD, Glatt SJ, Faraone SV (2018). Why is there selective subcortical vulnerability in ADHD? Clues from postmortem brain gene expression data. Mol. Psychiatry.

[55] Cloninger, C. R. & Svrakic, D. M. in *Kaplan and Sadock’s Comprehensive Textbook of Psychiatry* Vol. 1 (eds Sadock, B. J., Sadock, V. A. & Ruiz, P.) Ch. 26, 2126–2176 (Lippincott Williams & Wilkins, 2017).

[56] Cloninger CR (1994). Temperament and personality. Curr. Opin. Neurobiol..

[57] Corr PJ, Kumari V, Wilson GD, Checkley S, Gray JA (1997). Harm Avoidance and affective modulation of the startle reflex: a replication. Personal. Individ. Differences.

[58] Corr PJ, Pickering AD, Gray JA (1995). Personality and reinforcement in associative and instrumental learning. Personal. Individ. Differences.

[59] Pezawas L (2005). 5-HTTLPR polymorphism impacts human cingulate-amygdala interactions: a genetic susceptibility mechanism for depression. Nat. Neurosci..

[60] Houillon A (2013). The effect of novelty on reinforcement learning. Prog. Brain Res..

[61] Krebs RM, Schott BH, Duzel E (2009). Personality traits are differentially associated with patterns of reward and novelty processing in the human substantia nigra/ventral tegmental area. Biol. Psychiatry.

[62] Gusnard DA (2003). Persistence and brain circuitry. Proc. Natl Acad. Sci. USA.

[63] Cloninger CR, Zohar AH, Hirschmann S, Dahan D (2012). The psychological costs and benefits of being highly persistent: personality profiles distinguish mood disorders from anxiety disorders. J. Affect. Disord..

[64] Yang TT (2009). Adolescent subgenual anterior cingulate activity is related to harm avoidance. Neuroreport.

[65] Paulus MP, Rogalsky C, Simmons A, Feinstein JS, Stein MB (2003). Increased activation in the right insula during risk-taking decision making is related to harm avoidance and neuroticism. Neuroimage.

[66] Naghavi HR, Lind J, Nilsson LG, Adolfsson R, Nyberg L (2009). Personality traits predict response to novel and familiar stimuli in the hippocampal region. Psychiatry Res..

[67] Lei X (2014). Fiber connectivity between the striatum and cortical and subcortical regions is associated with temperaments in Chinese males. Neuroimage.

[68] Tost H (2010). A common allele in the oxytocin receptor gene (OXTR) impacts prosocial temperament and human hypothalamic-limbic structure and function. Proc. Natl Acad. Sci. USA.

[69] Hornowska E (2011). Cloninger’s psychobiological model of personality and Strelau’s regulative theory of temperament–analysis of their associations in a Polish sample. Pol. Psychological Bull..

[70] Windle M (1992). Temperament and social support in adolescence: Interrelations with depressive symptoms and delinquent behaviors. J. Youth Adolesc..

[71] Puttonen, S. *Common Elements of Five Temperament Models*. MA thesis, University of Helsinki (2005).

[72] Rowe DC, Plomin R (1977). Temperament in early childhood. J. Pers. Assess..

[73] Kitamura T (2014). Emotionality Activity Sociability and Impulsivity (EASI) Survey: psychometric properties and assessment of the Japanese version. Psychol. Behav. Sci..

[74] Buss, A. H. & Plomin, R. *Temperament: Early Developing Personality Traits*. (Lawrence Erlbaum, 1984).

[75] Thomas A, Chess S, Birch HG (1970). The origin of personality. Sci. Am..

[76] Thomas, A., Chess, S. & Birch, H. G. *Temperament and Behavior Disorders in Children*. (New York University Press, 1968).

[77] Carey WB, McDevitt SC (1978). Stability and change in individual temperament diagnoses from infancy to early childhood. J. Am. Acad. Child Psychiatry.

[78] Prior M (1992). Childhood temperament. J. Child Psychol. Psychiatry.

[79] Mathiesen KS, Tambs K (1999). The EAS temperament questionnaire–factor structure, age trends, reliability, and stability in a Norwegian sample. J. Child Psychol. Psychiatry.

[80] Rende RD (1993). Longitudinal relations between temperament traits and behavioral syndromes in middle childhood. J. Am. Acad. Child Adolesc. Psychiatry.

[81] Westen D, Shedler J (2000). A prototype matching approach to diagnosing personality disorders: toward DSM-V. J. Pers. Disord..

[82] Evans DE, Rothbart MK (2007). Developing a model for adult temperament. J. Res. Personal..

[83] Zuckerman M, Cloninger CR (1996). Relationship between Cloninger’s, Zuckerman’s, and Eysenck’s dimensions of personality. Personal. Individ. Differences.

[84] Cloninger CR (2010). Personality and temperament: New and alternative perspectives. Focus (Am. Psychiatr. Publ.).

[85] Hopwood CJ, Donnellan MB (2010). How should the internal structure of personality inventories be evaluated?. Pers. Soc. Psychol. Rev..

[86] Ropovik I (2015). A cautionary note on testing latent variable models. Front Psychol..

[87] Hayes AM, Laurenceau JP, Feldman G, Strauss JL, Cardaciotto L (2007). Change is not always linear: the study of nonlinear and discontinuous patterns of change in psychotherapy. Clin. Psychol. Rev..

[88] Lemery-Chalfant K, Doelger L, Goldsmith HH (2008). Genetic relations between effortful and attentional control and symptoms of psychopathology in middle childhood. Infant Child Dev..

[89] Tulving E (2001). Episodic memory and common sense: how far apart?. Philos. Trans. R. Soc. Lond., B Biol. Sci..

[90] Cloninger C. Robert, Zwir Igor (2018). What is the natural measurement unit of temperament: single traits or profiles?. Philosophical Transactions of the Royal Society B: Biological Sciences.

[91] Cloninger CR (1986). A unified biosocial theory of personality and its role in the development of anxiety states. Psychiatr. Dev..

[92] Cloninger CR (1987). A systematic method for clinical description and classification of personality variants. A proposal. Arch. Gen. Psychiatry.

[93] Hansenne M, Ansseau M (1998). Catecholaminergic function and temperament in major depressive disorder: a negative report. Psychoneuroendocrinology.

[94] Cloninger CR, Gilligan SB (1987). Neurogenetic mechanisms of learning: a phylogenetic perspective. J. Psychiatr. Res.

[95] Cloninger, C. R. & Kedia, S. in *The Origins of Cooperation and Altruism Developments in Primatology: Progress and Prospects* (eds Sussman, R. W. & Cloninger, C. R.) 63–110 (Springer, 2011).

[96] Van Schuerbeek P, Baeken C, De Raedt R, De Mey J, Luypaert R (2011). Individual differences in local gray and white matter volumes reflect differences in temperament and character: a voxel-based morphometry study in healthy young females. Brain Res..

[97] Gardini S, Cloninger CR, Venneri A (2009). Individual differences in personality traits reflect structural variance in specific brain regions. Brain Res. Bull..

[98] Povinelli, D. J. *Folk Physics for Apes: the Chimpanzee’s Theory of How the World Works*. (Oxford University Press, 2000).

[99] Ullman MT (2001). A neurocognitive perspective on language: the declarative/procedural model. Nat. Rev. Neurosci..

[100] Lee AC, Robbins TW, Graham KS, Owen AM (2002). “Pray or Prey?” dissociation of semantic memory retrieval from episodic memory processes using positron emission tomography and a novel homophone task. Neuroimage.

[101] Levine B (2004). The functional neuroanatomy of episodic and semantic autobiographical remembering: a prospective functional MRI study. J. Cogn. Neurosci..

[102] Finn AS (2016). Developmental dissociation between the maturation of procedural memory and declarative memory. J. Exp. Child Psychol..

[103] Nissen MJ, Knopman DS, Schacter DL (1987). Neurochemical dissociation of memory systems. Neurology.

[104] Graham KS, Simons JS, Pratt KH, Patterson K, Hodges JR (2000). Insights from semantic dementia on the relationship between episodic and semantic memory. Neuropsychologia.

[105] Svoboda E, McKinnon MC, Levine B (2006). The functional neuroanatomy of autobiographical memory: a meta-analysis. Neuropsychologia.

[106] Fink A (2018). Modulation of resting-state network connectivity by verbal divergent thinking training. Brain Cogn..

[107] Zwir, I. et al. Uncovering the complex genetics of human personality: response from authors on the PGMRA Model. *Mol. Psychiatry*, 10.1038/s4138-019-0399-z (2019). 10.1038/s41380-019-0399-z PMC7515846 30886336

[108] Keller MC, Coventry WL, Heath AC, Martin NG (2005). Widespread evidence for non-additive genetic variation in Cloninger’s and Eysenck’s Personality Dimensions using a Twin Plus Sibling Design. Behav. Genet..

[109] Eaves LJ, Heath AC, Neale MC, Hewitt JK, Martin NG (1998). Sex differences and non-additivity in the effects of genes on personality. Twin Res..

[110] Eaves LJ (1999). Comparing the biological and cultural inheritance of personality and social attitudes in the Virginia 30,000 study of twins and their relatives. Twin Res..

[111] Lake RI, Eaves LJ, Maes HH, Heath AC, Martin NG (2000). Further evidence against the environmental transmission of individual differences in neuroticism from a collaborative study of 45,850 twins and relatives on two continents. Behav. Genet.

[112] Finkel D, McGue M (1997). Sex differences and nonadditivity in heritability of the Multidimensional Personality Questionnaire Scales. J. Pers. Soc. Psychol..

[113] Verweij KJ (2012). Maintenance of genetic variation in human personality: testing evolutionary models by estimating heritability due to common causal variants and investigating the effect of distant inbreeding. Evolution.

[114] Vinkhuyzen AA (2012). Common SNPs explain some of the variation in the personality dimensions of neuroticism and extraversion. Transl. Psychiatry.

[115] Power RA, Pluess M (2015). Heritability estimates of the Big Five personality traits based on common genetic variants. Transl. Psychiatry.

[116] Genetics of Personality, C. (2015). Meta-analysis of genome-wide association studies for neuroticism, and the polygenic association with major depressive disorder. JAMA Psychiatry.

[117] Smith DJ (2016). Genome-wide analysis of over 106000 individuals identifies 9 neuroticism-associated loci. Mol. Psychiatry.

[118] Docherty AR (2016). SNP-based heritability estimates of the personality dimensions and polygenic prediction of both neuroticism and major depression: findings from CONVERGE. Transl. Psychiatry.

[119] Lo MT (2017). Genome-wide analyses for personality traits identify six genomic loci and show correlations with psychiatric disorders. Nat. Genet.

[120] Okbay A (2016). Genetic variants associated with subjective well-being, depressive symptoms, and neuroticism identified through genome-wide analyses. Nat. Genet.

[121] Luciano M (2018). Association analysis in over 329,000 individuals identifies 116 independent variants influencing neuroticism. Nat. Genet.

[122] Nagel M (2018). Meta-analysis of genome-wide association studies for neuroticism in 449,484 individuals identifies novel genetic loci and pathways. Nat. Genet.

[123] van den Berg SM (2016). Meta-analysis of genome-wide association studies for extraversion: findings from the genetics of personality consortium. Behav. Genet..

[124] Zwir I (2005). Dissecting the PhoP regulatory network of *Escherichia coli* and Salmonella enterica. Proc. Natl Acad. Sci. USA.

[125] Zwir I, Huang H, Groisman EA (2005). Analysis of differentially-regulated genes within a regulatory network by GPS genome navigation. Bioinformatics.

[126] Arnedo J (2015). Decomposition of brain diffusion imaging data uncovers latent schizophrenias with distinct patterns of white matter anisotropy. Neuroimage.

[127] Arnedo J (2015). Uncovering the hidden risk architecture of the schizophrenias: confirmation in three independent genome-wide association studies. Am. J. Psychiatry.

[128] Arnedo J (2013). PGMRA: a web server for (phenotype x genotype) many-to-many relation analysis in GWAS. Nucleic Acids Res..

[129] Raitakari OT (2008). Cohort profile: the cardiovascular risk in Young Finns Study. Int J. Epidemiol..

[130] Cook TB (2015). Latent infection with *Toxoplasma gondii*: association with trait aggression and impulsivity in healthy adults. J. Psychiatr. Res..

[131] Gombojav B (2013). The Healthy Twin Study, Korea updates: resources for omics and genome epidemiology studies. Twin Res. Hum. Genet..

[132] Sung J (2006). Healthy Twin: a twin-family study of Korea—protocols and current status. Twin Res. Hum. Genet.

[133] Deb, K. *Multi-objective Optimization Using Evolutionary Algorithms*. 1st edn, 497 (John Wiley & Sons, 2001).

[134] Luscher C, Malenka RC (2012). NMDA receptor-dependent long-term potentiation and long-term depression (LTP/LTD). Cold Spring Harb. Perspect. Biol..

[135] Peng S, Zhang Y, Zhang J, Wang H, Ren B (2010). ERK in learning and memory: a review of recent research. Int J. Mol. Sci..

[136] Tronson NC, Corcoran KA, Jovasevic V, Radulovic J (2012). Fear conditioning and extinction: emotional states encoded by distinct signaling pathways. Trends Neurosci..

[137] Brems B (2011). Spontaneous decisions and operant conditioning in fruit flies. Behav. Process..

[138] Rapanelli M, Frick LR, Zanutto BS (2009). Differential gene expression in the rat hippocampus during learning of an operant conditioning task. Neuroscience.

[139] Atkins CM, Selcher JC, Petraitis JJ, Trzaskos JM, Sweatt JD (1998). The MAPK cascade is required for mammalian associative learning. Nat. Neurosci..

[140] Sakai N, Ohno H, Tomioka M, Iino Y (2017). The intestinal TORC2 signaling pathway contributes to associative learning in *Caenorhabditis elegans*. PLoS ONE.

[141] Diamond DM, Park CR, Campbell AM, Woodson JC (2005). Competitive interactions between endogenous LTD and LTP in the hippocampus underlie the storage of emotional memories and stress-induced amnesia. Hippocampus.

[142] Rapanelli M, Frick LR, Zanutto BS (2011). Learning an operant conditioning task differentially induces gliogenesis in the medial prefrontal cortex and neurogenesis in the hippocampus. PLoS ONE.

[143] Rapanelli M, Frick LR, Bernardez-Vidal M, Zanutto BS (2013). Different MK-801 administration schedules induce mild to severe learning impairments in an operant conditioning task: role of buspirone and risperidone in ameliorating these cognitive deficits. Behav. Brain Res..

[144] Swanson JM (2007). Etiologic subtypes of attention-deficit/hyperactivity disorder: brain imaging, molecular genetic and environmental factors and the dopamine hypothesis. Neuropsychol. Rev..

[145] Ramanathan C (2018). mTOR signaling regulates central and peripheral circadian clock function. PLoS Genet.

[146] Mendoza MC, Er EE, Blenis J (2011). The Ras-ERK and PI3K-mTOR pathways: cross-talk and compensation. Trends Biochem Sci..

[147] Fruman DA (2017). The PI3K pathway in human disease. Cell.

[148] Steelman LS (2011). Roles of the Raf/MEK/ERK and PI3K/PTEN/Akt/mTOR pathways in controlling growth and sensitivity to therapy-implications for cancer and aging. Aging (Albany NY).

[149] Lee Myoungsook, Sorn Sungbin Richard, Lee Yunkyoung, Kang Inhae (2019). Salt Induces Adipogenesis/Lipogenesis and Inflammatory Adipocytokines Secretion in Adipocytes. International Journal of Molecular Sciences.

[150] Liong S, Barker G, Lappas M (2018). Placental ras regulates inflammation associated with maternal obesity. Mediators Inflamm..

[151] Hintsanen M (2011). Negative emotionality, activity, and sociability temperaments predicting long-term job strain and effort-reward imbalance: a 15-year prospective follow-up study. J. Psychosom. Res..

[152] Wessman J (2012). Temperament clusters in a normal population: implications for health and disease. PLoS One.

[153] Hintsanen M (2009). Cloninger’s temperament traits and preclinical atherosclerosis: the Cardiovascular Risk in Young Finns Study. J. Psychosom. Res..

[154] Chan EY, Stang SL, Bottorff DA, Stone JC (1999). Hypothermic stress leads to activation of Ras-Erk signaling. J. Clin. Invest.

[155] Molina JR, Adjei AA (2006). The Ras/Raf/MAPK pathway. J. Thorac. Oncol..

[156] Yoshihara T (2013). Heat stress activates the Akt/mTOR signalling pathway in rat skeletal muscle. Acta Physiol. (Oxf.).

[157] Kakigi R (2011). Heat stress enhances mTOR signaling after resistance exercise in human skeletal muscle. J. Physiol. Sci..

[158] Gomes AP, Adjei AA (2015). A nexus for cellular homeostasis: the interplay between metabolic and signal transduction pathways. Curr. Opin. Biotechnol..

[159] Zhang K (2014). Light-mediated kinetic control reveals the temporal effect of the Raf/MEK/ERK pathway in PC12 cell neurite outgrowth. PLoS ONE.

[160] Krishnamurthy, V. V. et al. Light-mediated reversible modulation of the mitogen-activated protein kinase pathway during cell differentiation and xenopus embryonic development. *J. Vis. Exp.*10.3791/55823 (2017). 10.3791/55823 PMC5608446 28654043

[161] Caraglia M (2005). Electromagnetic fields at mobile phone frequency induce apoptosis and inactivation of the multi-chaperone complex in human epidermoid cancer cells. J. Cell Physiol..

[162] Pall ML (2016). Microwave frequency electromagnetic fields (EMFs) produce widespread neuropsychiatric effects including depression. J. Chem. Neuroanat..

[163] Altun G (2017). Protective effects of melatonin and omega-3 on the hippocampus and the cerebellum of adult Wistar albino rats exposed to electromagnetic fields. J. Microsc Ultrastruct..

[164] Buckner CA, Buckner AL, Koren SA, Persinger MA, Lafrenie RM (2018). Exposure to a specific time-varying electromagnetic field inhibits cell proliferation via cAMP and ERK signaling in cancer cells. Bioelectromagnetics.

[165] Rouleau N, Dotta BT (2014). Electromagnetic fields as structure-function zeitgebers in biological systems: environmental orchestrations of morphogenesis and consciousness. Front Integr. Neurosci..

[166] Singh A (2019). Personalized repetitive transcranial magnetic stimulation temporarily alters default mode network in healthy subjects. Sci. Rep..

[167] Siddiqi SH, Chockalingam R, Cloninger CR, Lenze EJ, Cristancho P (2016). Use of the temperament and character inventory to predict response to repetitive transcranial magnetic stimulation for major depression. J. Psychiatr. Pr..

[168] Wang, J. Q. & Mao, L. The ERK pathway: molecular mechanisms and treatment of depression. *Mol. Neurobiol*. (2019). 10.1007/s12035-019-1524-3 PMC6684449 30737641

[169] Schliess F, Richter L, vom Dahl S, Haussinger D (2006). Cell hydration and mTOR-dependent signalling. Acta Physiol..

[170] Vitalini MW (2007). Circadian rhythmicity mediated by temporal regulation of the activity of p38 MAPK. Proc. Natl Acad. Sci. USA.

[171] Dubbelhuis PF, Meijer AJ (2002). Hepatic amino acid-dependent signaling is under the control of AMP-dependent protein kinase. FEBS Lett..

[172] Howell JJ (2017). Metformin inhibits hepatic mTORC1 signaling via dose-dependent mechanisms involving AMPK and the TSC complex. Cell Metab..

[173] Wright DC (2007). Exercise-induced mitochondrial biogenesis begins before the increase in muscle PGC-1alpha expression. J. Biol. Chem..

[174] Wright DC, Geiger PC, Han DH, Jones TE, Holloszy JO (2007). Calcium induces increases in peroxisome proliferator-activated receptor gamma coactivator-1alpha and mitochondrial biogenesis by a pathway leading to p38 mitogen-activated protein kinase activation. J. Biol. Chem..

[175] Perry CGR (2017). Mitochondrial adaptations to exercise in human skeletal muscle: a possible role for cristae density as a determinant of muscle fitness. J. Physiol..

[176] Miao S, Liu Y, Zhang L, Shan M, Miao Z (2018). Effects of MAPK/ERK pathway on learning and memory in sleep deprivation rats. Int. J. Clin. Exp. Med..

[177] Perogamvros L (2015). Increased reward-related behaviors during sleep and wakefulness in sleepwalking and idiopathic nightmares. PLoS ONE.

[178] Mikhail Cyril, Vaucher Angélique, Jimenez Sonia, Tafti Mehdi (2017). ERK signaling pathway regulates sleep duration through activity-induced gene expression during wakefulness. Science Signaling.

[179] Randler C, Saliger L (2011). Relationship between morningness-eveningness and temperament and character dimensions in adolescents. Personal. Individ. Differences.

[180] Johansson C (2003). Circadian clock-related polymorphisms in seasonal affective disorder and their relevance to diurnal preference. Neuropsychopharmacology.

[181] Chotai J (2005). Novelty seekers and summer-borns are likely to be low in morningness. Eur. Psychiatry.

[182] Chotai J, Joukamaa M, Taanila A, Lichtermann D, Miettunen J (2009). Novelty seeking among adult women is lower for the winter borns compared to the summer borns: replication in a large Finnish birth cohort. Compr. Psychiatry.

[183] Van Schuerbeek P, Baeken C, Luypaert R, De Raedt R, De Mey J (2014). Does the amygdala response correlate with the personality trait ‘harm avoidance’ while evaluating emotional stimuli explicitly?. Behav. Brain Funct..

[184] Lander ES (2019). Adopt a moratorium on heritable genome editing. Nature.

[185] Bleakley CM, Bieuzen F, Davison GW, Costello JT (2014). Whole-body cryotherapy: empirical evidence and theoretical perspectives. Open Access J. Sports Med..

[186] Costello, J. T. et al. Whole-body cryotherapy (extreme cold air exposure) for preventing and treating muscle soreness after exercise in adults. *Cochrane Database SystRev* CD010789, 10.1002/14651858.CD010789.pub2 (2015). 10.1002/14651858.CD010789.pub2 PMC9579836 26383887

[187] Shui S, Wang X, Chiang JY, Zheng L (2015). Far-infrared therapy for cardiovascular, autoimmune, and other chronic health problems: a systematic review. Exp. Biol. Med. (Maywood).

[188] Oldham MA, Ciraulo DA (2014). Bright light therapy for depression: a review of its effects on chronobiology and the autonomic nervous system. Chronobiol. Int.

[189] Reichborn-Kjennerud T, Lingjaerde O (1996). Response to light therapy in seasonal affective disorder: personality disorders and temperament as predictors of outcome. J. Affect Disord..

[190] Nigg CR (1999). Stages of change across ten health risk behaviors for older adults. Gerontologist.

[191] Prochaska JO (2004). Multiple risk expert systems interventions: impact of simultaneous stage-matched expert system interventions for smoking, high-fat diet, and sun exposure in a population of parents. Health Psychol..

[192] Kobau R, Sniezek J, Zack MM, Lucas RE, Adam B (2010). Well-being assessment: An evaluation of well-being scales for public health and population stimates of well-being among US adults. Appl. Psychol..

[193] Krueger M, Costello JT, Achtzehn S, Dittmar KH, Mester J (2019). Whole-body cryotherapy (−110 degrees C) following high-intensity intermittent exercise does not alter hormonal, inflammatory or muscle damage biomarkers in trained males. Cytokine.

[194] Polidori G (2018). Should whole body cryotherapy sessions be differentiated between women and men? A preliminary study on the role of the body thermal resistance. Med Hypotheses.

[195] Cloninger CR, Cloninger KM (2013). People create health: Effective health promotion is a creative process. *International*. J. Pers.-centered Med..

[196] Mirowsky J, Ross CE (1998). Education, personal control, lifestyle and health: a human capital hypothesis. Res. Aging.

[197] Hayes SA, Orsillo SM, Roemer L (2010). Changes in proposed mechanisms of action during an acceptance-based behavior therapy for generalized anxiety disorder. Behav. Res Ther..

[198] Cloninger CR (2013). Person-centered health promotion in chronic disease. Int. J. Pers.-centered Med..

[199] Cloninger CR, Cloninger KM (2011). Person-centered therapeutics. Int. J. Pers.-centered Med..

