# Supplementary material for: The complex genetics and biology of human temperament: a review of traditional concepts in relation to new molecular findings
Source: Transl Psychiatry. 2019 Nov 11;9:290. doi: 10.1038/s41398-019-0621-4 (PMC6848211; doi:10.1038/s41398-019-0621-4)
Supplement: Supplementary file 1 — Supplementary Information [file 41398_2019_621_MOESM1_ESM.docx]

**Supplementary Information**

**Outline**

A. Supplementary Tables 1-7

1. Four ancient temperament types based on analogy to

environmental perturbations of four seasons

2. Descriptions of high and low scorers on dimensions of

Temperament and Character Inventory

3. Joint factor analysis of four temperament inventories: temperaments as measured by TCI, FCB-TI, EAS, and

DOTS-R in the Young Finns Study

4. Distinguishing features of three subtypes or clusters of

temperament features of Thomas and Chess

5. Estimates of additive and non-additive heritability of

temperament and other personality traits based on

studies of extended families including twins

6. Estimates of additive heritability of temperament and other

personality traits explained by additive effects of SNPs

7. Distinguishing features of three clusters of people with replicated

relationships between genotypic sets and temperament sets (see Table 5) using descriptors from TCI and from eight other descriptive models of temperament

B. Supplementary Figure 1

C. References for Supplementary Information

A. **Supplementary Tables**

**Supplemental Table 1**. Four ancient temperament types based on analogy to environmental perturbations of four seasons (based on Galen and Avicenna as described by Kant ^1^ and Cloninger and Svrakic ^2^)

| **Distinguishing Features** | **Sanguine**  **Subtype** | **Choleric**  **Subtype** | **Melancholic**  **Subtype** | **Phlegmatic Subtype** |
| --- | --- | --- | --- | --- |
| Preferred  Seasonal Conditions | wet/hot  (spring) | dry/hot  (summer) | dry/cold  (autumn) | wet/cold  (winter) |
| Emotional Style | happy | angry | sad | peaceful |
| Personality Features | stable and active extravert | unstable and active  extravert | unstable and inactive  introvert | stable and inactive  introvert |
| Valence of Mood | positive | negative | negative | positive |
| Intensity of Arousal | low | high | high | low |
| Sensitivity to Rewards | high | variable | variable | low |
| Sensitivity to Novelty | average | high | average | low |
| Sensitivity to Signals of Punishment | low | average | high | average |

**Supplemental Table 2.** Description of high and low scorers on dimensions of the Temperament and Character Inventory ^3^

| **TCI Scales** | **TCI Subscales** | **High Scorers** | **Low Scorers** |
| --- | --- | --- | --- |
| **Novelty Seeking** |  |  |  |
|  | NS1 excitability | exploratory | reserved |
|  | NS2 impulsivity | impulsive | rigid |
|  | NS3 extravagance | extravagant | thrift |
|  | NS4 disorderly | rule-breaking | orderly |
| **Harm Avoidance** |  |  |  |
|  | HA1 pessimism | pessimistic | optimistic |
|  | HA2 fearfulness | fearful | risk-taking |
|  | HA3 shyness | shy | outgoing |
|  | HA4 fatigability | fatigable | vigorous |
| **Reward Dependence** |  |  |  |
|  | RD1 sentimentality | sentimental | objective |
|  | RD2 openness | warm | aloof |
|  | RD3 attachment | friendly | detached |
|  | RD4 dependent | approval-seeking | independent |
| **Persistence** |  |  |  |
|  | PS1 eagerness | enthusiastic | hesitant |
|  | PS2 hard-working | determined | spoiled |
|  | PS3 ambition | ambitious | underachieving |
|  | PS4 perfectionism | perfectionistic | pragmatic |
| **Self-directedness** |  |  |  |
|  | SD1 responsibility | responsible | blaming |
|  | SD2 purposefulness | purposeful | aimless |
|  | SD3 resourcefulness | resourceful | helpless |
|  | SD4 self-acceptance | unpretentious | pretentious |
|  | SD5 self-actualizing | self-actualizing | unfulfilled |
| **Cooperativeness** |  |  |  |
|  | CO1 social tolerance | tolerant | prejudiced |
|  | CO2 empathy | empathetic | self-centered |
|  | CO3 helpfulness | considerate | hostile |
|  | CO4 compassion | forgiving | revengeful |
|  | CO5 conscience | principled | opportunistic |
| **Self-transcendence** |  |  |  |
|  | ST1 self-forgetfulness | engaged | self-concerned |
|  | ST2 transpersonal  identification | joyfully connected  altruistic | separate  individualistic |
|  | ST3 spiritual  acceptance | faithful | skeptical |
|  | ST4 contemplation | contemplative | conventional |
|  | ST5 idealism | idealistic | cynical |

**Supplementary Table 3**. Joint factor analysis of four temperament inventories: temperaments as measured by TCI, EAS, FCB-TI, and DOTS-R in 2106 participants aged 20 to 35 in the Young Finns Study ^4^

| Temperament measures | Factor 1 | Factor 2 | Factor 3 | Factor 4 | Factor 5 | Factor 6 |
| --- | --- | --- | --- | --- | --- | --- |
| **TCI** |  |  |  |  |  |  |
| Harm Avoidance | **- .66** | - .18 |  | - .24 | - .16 | - .19 |
| Novelty Seeking |  | .12 | - .12 | **.53** | - .33 | .37 |
| Reward Dependence | - .32 | **.69** |  |  |  |  |
| Persistence |  |  |  | .14 | **.68** |  |
| **FCB-TI** |  |  |  |  |  |  |
| Emotional Reactivity | **- .85** |  |  |  | - .11 |  |
| Briskness | .36 |  |  | .17 | .18 | .20 |
| Sensory Sensitivity |  |  |  |  | .14 | .19 |
| Activity | .27 | .44 |  | .45 |  |  |
| Perseverance | **- .69** | .14 |  |  | .11 |  |
| Endurance | **.63** |  | - .11 |  | .18 |  |
| **EAS** |  |  |  |  |  |  |
| Negative Emotionality | **- .75** | - .26 |  | .24 |  |  |
| Activity |  |  |  | **.50** | .30 | .11 |
| Sociability |  | **.80** |  | .13 |  |  |
| **DOTS-R** |  |  |  |  |  |  |
| Activity-general |  |  | **.67** |  |  | - .17 |
| Activity-sleep |  |  | - .14 | .24 |  |  |
| Approach vs withdrawal | .26 | .47 |  | .23 |  | .27 |
| Flexibility vs rigidity | .34 |  | - .19 | - .12 |  | **.55** |
| Mood Quality | .23 | .45 |  |  |  | .20 |
| Rhythmicity--sleep |  |  | **.73** |  |  |  |
| Rhythmicity-eating |  |  | **.74** |  |  |  |
| Rhythmicity-daily habits |  |  | **.75** |  |  |  |
| Low Distractibility | .14 |  |  |  | .46 |  |
| Persistence |  | .10 |  | - .16 | **.58** | .11 |

**Supplementary Table 4**. Distinguishing features of three subtypes or clusters of temperament from 2 months to 10 years (adapted from Thomas, Chess, Birch, 1970 ^5^; Thomas, Chess, 1977 ^6^; Carey and McDevitt, 1978 ^7^)

| **Dimensions of Temperament** | **Easy**  **Subtype** | **Difficult Subtype** | **Slow-to-Warm-Up**  **Subtype** |
| --- | --- | --- | --- |
| Activity Level | variable | variably high | low to moderate |
| Rhythmicity (sleep, eating, toileting) | highly regular | irregular | variable |
| Adaptability to changes in routine | rapid | slow | slow |
| Approachability | positive approach | withdrawal | initial withdrawal |
| Intensity of emotional arousal | low or mild | intense | mild |
| Valence of Mood | positive | negative | indifferent or slightly negative |

**Supplementary Table 5**. Estimates of additive and non-additive heritability of temperament and other personality traits based on studies of extended families including twins*

| **Trait** | **additive**  **heritability**  **(h^2^) %** | **non-additive**  **heritability**  **(i^2^) %** | **Reference** |
| --- | --- | --- | --- |
| TCI |  |  |  |
| Harm  Avoidance | 27 | 28 | Keller et al, 2005 ^8^ |
| Novelty  Seeking | 20 | 35 | Keller et al, 2005 |
| Reward  Dependence | 32.5 | 21 | Keller et al, 2005 |
| Persistence | 20 | 35 | Keller et al, 2005 |
| EPQ |  |  |  |
| Neuroticism | 34 | 17.5 | Keller et al, 2005 |
|  | 11.3 | 38.9 | Eaves et al, 1998 ^9^ |
|  | 23.1 | 7.9 | Eaves et al, 1999 ^10^ |
|  | 42 | 15 | Lake et al, 2000 ^11^ |
| Extraversion |  |  |  |
|  | 33 | 24 | Keller et al, 2005 |
|  | 26.1 | 21.8 | Eaves et al, 1998 |
|  | 22.1 | 24.3 | Eaves et al, 1999 |
| MPQ |  |  |  |
| Negative  Emotionality | 27 | 18 | Finkel & McGue, 1997 ^12^ |
| Positive  Emotionality | 30 | 20.5 | Finkel & McGue, 1997 |
| Constraint | 33.5 | 18 | Finkel & McGue, 1997 |

* men and women were combined due to non-significant differences.

The broad heritability is the sum of the additive and non-additive heritability.

**Supplementary Table 6**. Estimates of additive heritability of temperament and other personality traits explained by additive effects of SNPs

| **Trait** | **Inventory** | **Sample**  **Size**  **(n)** | **additive SNP heritability**  **% (SE)** | **Reference** |
| --- | --- | --- | --- | --- |
| Temperament |  |  |  |  |
| Harm  Avoidance | TCI | 8,613 | 6.6 (3.7) | Verweij et al, 2012 ^13^ |
| Novelty  Seeking | TCI | 8,620 | 9.9 (3.6) | Verweij et al, 2012 |
| Reward  Dependence | TCI | 8,606 | 4.2 (3.6) | Verweij et al, 2012 |
| Persistence | TCI | 8,618 | 8.1 (3.7) | Verweij et al, 2012 |
| Neuroticism |  |  |  |  |
|  | EPQ | 12,000 | 6.0 (3.0) | Vinkhuyzen et al, 2012 ^14^ |
|  | NEO-IPIP | 5,011 | 15.0 (8.0) | Power & Pleuss, 2015 ^15^ |
|  | IRT harmonized | 63,611 | 14.7 (5.5)  15.7 (8.3) | de Moor et al, 2015 ^16^ |
|  | EPQ | 91,370 | 14.1 (1.5) | Smith et al, 2016 ^17^ |
|  | EPQ | 9,633 | 10.0 (3.0) | Docherty et al, 2016 ^18^ |
|  | Big Five | 4,728 | 0 (5.0) | Docherty et al, 2016 |
|  | EPQ, NEO-FFI | 59,176 | 11.9 (1.6) | Lo et al, 2017 ^19^ |
|  | EPQ, NEO | 170,911 | 9.1 (0.7) | Okbay et al, 2016 ^20^ |
|  | EPQ | 329,821 | 10.8 (0.5) | Luciano et al, 2018 ^21^ |
|  | EPQ, NEO, NEO-FFI | 449,484 | 10.8 (0.3) | Nagel et al, 2018 ^22^ |
| Extraversion |  |  |  |  |
|  | EPQ | 12,000 | 12.0 (3.0) | Virkhuyzen et al, 2012 |
|  | NEO-IPIP | 5,011 | 8.0 (8.0) | Power & Pleuss, 2015 |
|  | Big Five | 4,728 | 0 (6.0) | Docherty et al, 2016 |
|  | IRT harmonized | 3,597  3,369 | 4.9 (0.8) | van den Berg et al, 2016 ^23^ |
|  | EPQ, NEO-FFI | 59,176 | 18.1 (1.0) | Lo et al, 2017 |
| Openness |  |  |  |  |
|  | NEO-IPIP | 5,011 | 21.0 (8.0) | Power & Pleuss, 2015 |
|  | Big Five | 4,728 | 3.0 (6.0) | Docherty et al, 2016 |
|  | NEO-FFI | 59,176 | 10.7 (0.9) | Lo et al, 2017 |
| Agreeability | NEO-FFI | 59,176 | 8.5 (0.9) | Lo et al, 2017 |
|  | NEO-IPIP | 5,011 | 0 (8.0) | Power & Pleuss, 2015 |
|  | Big Five | 4,728 | 10.0 (6.0) | Docherty et al, 2016 |
| Conscientiousness | NEO-FFI | 59,176 | 9.6 (0.9) | Lo et al, 2017 |
|  | NEO-IPIP | 5,011 | 1.0 (8.0) | Power & Pleuss, 2015 |
|  | Big Five | 4,728 | 5.0 (6.0) | Docherty et al, 2016 |

**Supplementary Table S7**. Distinguishing features of three clusters of people with replicated relationships between genotypic sets and temperament sets (see Table 5) using descriptors from TCI and from eight other descriptive models of temperament.*

| Dimensions of Temperament  (and character) | **Reliable**  **Cluster** | **Sensitive**  **Cluster** | **Antisocial**  **Cluster** |
| --- | --- | --- | --- |
| **Cloninger TCI** |  |  |  |
| Harm Avoidance | Low | High | variable |
| Novelty Seeking | Low | High | High |
| Reward  Dependence | High | High | Low |
| Persistence | High | variable | low |
| (Self-direction) | variable | variable | variable |
| (Cooperation) | variable | variable | variable |
| (Transcendence) | variable | variable | variable |
|  |  |  |  |
| **Thomas & Chess** | **"easy"** | **"difficult"** | **"slow to warm up"** |
| Activity Level | variable | variably high | low to moderate |
| Rhythmicity  (sleep, eating,  toileting) | highly regular | irregular | variable |
| Adaptability to  changes in  routine | rapid | slow | slow |
| Approachability | positive approach | withdrawal | initial withdrawal |
| Intensity of  emotional  arousal | low or mild | intense | mild |
| Valence of  Mood | positive | negative | indifferent or slightly negative |
|  |  |  |  |
| **Strelau** **FCB-TI** |  |  |  |
| Emotional  Reactivity | low | high | variable |
| Briskness | high | low | variable |
| Sensory  Sensitivity | variable | high | variable |
| (Extravert) Activity | high | high | low |
| Perseverance | high | low | low |
| Endurance | high | low | high |
|  |  |  |  |
| **Buss-Plomin**  **EASI** & **CCTI** |  |  |  |
| (Negative)  Emotionality | Low | High | variable |
| (Motor) Activity | variable | variable | variable |
| Sociability | High | High | Low |
| Impulsivity | low | high | high |
| Shyness | low | high | variable |
| Soothability | high | low | low |
| Persistence | high | variable | low |
|  |  |  |  |
| **Rothbart CBQ** |  |  |  |
| Negative  Affectivity | low | high | variable |
| Extraversion | high | high | low |
| Effortful Control | high | low | low |
|  |  |  |  |
| **Eysenck PQ-R** |  |  |  |
| Neuroticism | low | high | variable |
| Extraversion | high | high | low |
| Psychoticism | low | low | high |
|  |  |  |  |
| **Zuckerman- KPQ** |  |  |  |
| Neuroticism | low | high | variable |
| Impulsive Sensation-Seeking | low | high | high |
| Sociability | high | high | low |
| (Persistent) Activity | high | low | low |
| (Hostility) | variable | variable | variable |
|  |  |  |  |
| **NEO-PI-R** |  |  |  |
| Neuroticism | low | high | variable |
| Extraversion | high | high | low |
| Openness | low | high | high |
| Conscientiousness | high | low | low |
| (Agreeability) | variable | variable | variable |

*Temperament profiles are based on Zwir et al, 2018 (Uncovering the complex genetics of human temperament), and the relations of TCI to other measurement systems from available data (Tables 2-4, Supplementary Table S5). Measured traits are rated "variable" with respect to temperament-associated genotypic sets if they are not correlated with any TCI temperament in Tables 2-4 (e.g., EAS motor activity, ZKPQ hostility, NEO agreeability). If measured traits are composites of both TCI temperament and self-regulatory character dimensions (e.g., Rothbart's effortful control and NEO conscientiousness that are correlated with both TCI Persistence and Self-directedness), they are expected to have some association with temperament-related genotypic sets on average but not consistently in every individual.

B. **Supplemental Figure 1**

Distribution of expression of genes associated with personality

(reproduced from Figure S9, Zwir et al, *Molecular Psychiatry* 2019, Three Genetic-Environmental Networks for Human Personality).


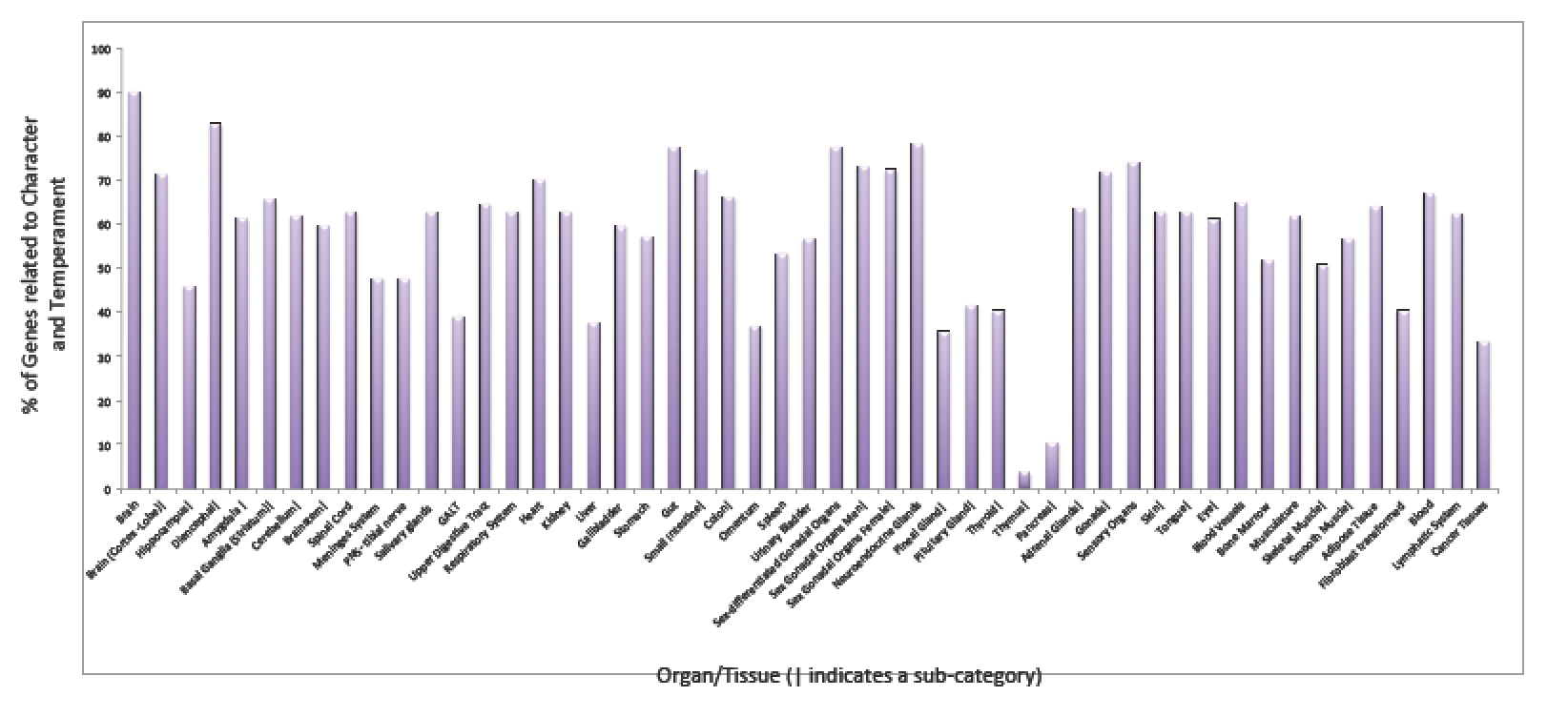


C. **References for Supplementary Information**

1 Kant, I. *Anthropology from a pragmatic point of view*. 1978 edn, (Southern Illinois University Press, 1797).

2 Cloninger, C. R. & Svrakic, D. M. in *Kaplan and Sadock's Comprehensive Textbook of Psychiatry* Vol. 1 (eds Benjamin J. Sadock, Virginia A. Sadock, & Pedro Ruiz) Ch. 26, 2126-2176 (Lippincott Williams & Wilkins, 2017).

3 Cloninger, C. R., Svrakic, D. M. & Przybeck, T. R. A psychobiological model of temperament and character. *Archives of General Psychiatry* **50**, 975-990 (1993).

4 Puttonen, S. *Common elements of five temperament models* PhD thesis, University of Helsinki, (2005).

5 Thomas, A., Chess, S. & Birch, H. G. The origin of personality. *Scientific American* **223**, 102-109, doi:10.1038/scientificamerican0870-102 (1970).

6 Thomas, A. & Chess, S. *Temperament and Development*. (Brunner/Mazel, 1977).

7 Carey, W. B. & McDevitt, S. C. Stability and change in individual temperament diagnoses from infancy to early childhood. *J Am Acad Child Psychiatry* **17**, 331-337 (1978).

8 Keller, M. C., Coventry, W. L., Heath, A. C. & Martin, N. G. Widespread evidence for non-additive genetic variation in Cloninger's and Eysenck's Personality Dimensions using a Twin Plus Sibling Design. *Behavior Genetics* **35**, 707-721, doi:10.1007/s10519-005-6041-7 (2005).

9 Eaves, L. J., Heath, A. C., Neale, M. C., Hewitt, J. K. & Martin, N. G. Sex differences and non-additivity in the effects of genes on personality. *Twin Res* **1**, 131-137 (1998).

10 Eaves, L. J. *et al.* Comparing the biological and cultural inheritance of personality and social attitudes in the Virginia 30,000 study of twins and their relatives. *Twin Research* **2**, 62-80 (1999).

11 Lake, R. I., Eaves, L. J., Maes, H. H., Heath, A. C. & Martin, N. G. Further evidence against the environmental transmission of individual differences in neuroticism from a collaborative study of 45,850 twins and relatives on two continents. *Behav Genet* **30**, 223-233 (2000).

12 Finkel, D. & McGue, M. Sex differences and nonadditivity in heritability of the Multidimensional Personality Questionnaire Scales. *J Pers Soc Psychol* **72**, 929-938 (1997).

13 Verweij, K. J. *et al.* Maintenance of genetic variation in human personality: testing evolutionary models by estimating heritability due to common causal variants and investigating the effect of distant inbreeding. *Evolution* **66**, 3238-3251, doi:10.1111/j.1558-5646.2012.01679.x (2012).

14 Vinkhuyzen, A. A. *et al.* Common SNPs explain some of the variation in the personality dimensions of neuroticism and extraversion. *Transl Psychiatry* **2**, e102, doi:10.1038/tp.2012.27 (2012).

15 Power, R. A. & Pluess, M. Heritability estimates of the Big Five personality traits based on common genetic variants. *Transl Psychiatry* **5**, e604, doi:10.1038/tp.2015.96 (2015).

16 Genetics of Personality, C. *et al.* Meta-analysis of Genome-wide Association Studies for Neuroticism, and the Polygenic Association With Major Depressive Disorder. *JAMA Psychiatry* **72**, 642-650, doi:10.1001/jamapsychiatry.2015.0554 (2015).

17 Smith, D. J. *et al.* Genome-wide analysis of over 106 000 individuals identifies 9 neuroticism-associated loci. *Mol Psychiatry* **21**, 1644, doi:10.1038/mp.2016.177 (2016).

18 Docherty, A. R. *et al.* SNP-based heritability estimates of the personality dimensions and polygenic prediction of both neuroticism and major depression: findings from CONVERGE. *Transl Psychiatry* **6**, e926, doi:10.1038/tp.2016.177 (2016).

19 Lo, M. T. *et al.* Genome-wide analyses for personality traits identify six genomic loci and show correlations with psychiatric disorders. *Nat Genet* **49**, 152-156, doi:10.1038/ng.3736 (2017).

20 Okbay, A. *et al.* Genetic variants associated with subjective well-being, depressive symptoms, and neuroticism identified through genome-wide analyses. *Nat Genet* **48**, 624-633, doi:10.1038/ng.3552 (2016).

21 Luciano, M. *et al.* Association analysis in over 329,000 individuals identifies 116 independent variants influencing neuroticism. *Nat Genet* **50**, 6-11, doi:10.1038/s41588-017-0013-8 (2018).

22 Nagel, M. *et al.* Meta-analysis of genome-wide association studies for neuroticism in 449,484 individuals identifies novel genetic loci and pathways. *Nat Genet* **50**, 920-927, doi:10.1038/s41588-018-0151-7 (2018).

23 van den Berg, S. M. *et al.* Meta-analysis of Genome-Wide Association Studies for Extraversion: Findings from the Genetics of Personality Consortium. *Behav Genet* **46**, 170-182, doi:10.1007/s10519-015-9735-5 (2016).
